# Characterization of *Pseudomonas aeruginosa* from subjects with diffuse panbronchiolitis

**DOI:** 10.1128/spectrum.00530-24

**Published:** 2024-10-08

**Authors:** Charles M. Met, Casey E. Hofstaedter, Ian P. O'Keefe, Hyojik Yang, Dina A. Moustafa, Matthew E. Sherman, Yohei Doi, David A. Rasko, Charles R. Sweet, Joanna B. Goldberg, Robert K. Ernst

**Affiliations:** 1 Department of Microbial Pathogenesis, University of Maryland – Baltimore, Baltimore, Maryland, USA; 2 Medical Scientist Training Program, University of Maryland – Baltimore, Baltimore, Maryland, USA; 3 Department of Biochemistry and Molecular Biology, University of Maryland – Baltimore, Baltimore, Maryland, USA; 4 Department of Pediatrics, Division of Pulmonary, Asthma, Cystic Fibrosis, and Sleep, Emory University School of Medicine, Atlanta, Georgia, USA; 5 Department of Medicine, University of Pittsburgh School of Medicine, Pittsburgh, Pennsylvania, USA; 6 Department of Microbiology and Immunology, Institute for Genome Sciences, University of Maryland - Baltimore, Baltimore, Maryland, USA; 7 Chemistry Department, USA Naval Academy, Annapolis, Maryland, USA; Universita degli Studi Roma Tre Dipartimento di Scienze, Rome, Italy

**Keywords:** *Pseudomonas aeruginosa*, diffuse panbronchiolitis, LPS, adaptation

## Abstract

Diffuse panbronchiolitis (DPB) is a rare, idiopathic inflammatory disease primarily diagnosed in East Asian populations. DPB is characterized by diffuse pulmonary lesions, inflammation of the respiratory bronchioles, and bacterial infections of the airway. Historically, sputum cultures reveal *Pseudomonas aeruginosa* in 22% of DPB patients, increasing to 60% after 4 years from disease onset. Although DPB patients have a known susceptibility to respiratory *P. aeruginosa* infections, as is observed in other chronic lung diseases such as cystic fibrosis (CF), the characterization of DPB *P. aeruginosa* strains is limited. In this study, we characterized 24 strains obtained from a cohort of DPB patients for traits previously associated with virulence, including growth, motility, antibiotic susceptibility, lipopolysaccharide structure, and genomic diversity. Our cohort of DPB *P. aeruginosa* strains exhibits considerable genomic variability when compared with isolates from people with cystic fibrosis chronically colonized with *P. aeruginosa* and acute *P. aeruginosa* infection isolates. Similar to CF, DPB *P. aeruginosa* strains produce a diverse array of modified lipid A structures. Antibiotic susceptibility testing revealed increased resistance to erythromycin, a representative agent of the macrolide antibiotics used to manage DPB patients. Differences in the O-antigen type among *P. aeruginosa* strains collected from these different backgrounds were also observed. Ultimately, the characterization of DPB *P. aeruginosa* strains highlights several unique qualities of *P. aeruginosa* strains collected from chronically diseased airways, underscoring the challenges in treating DPB, CF, and other obstructive respiratory disease patients with *P. aeruginosa* infections.

**IMPORTANCE:**

Diffuse panbronchiolitis (DPB), a chronic lung disease characterized by persistent *P. aeruginosa* infection, serves as an informative comparator to more common chronic lung diseases, such as cystic fibrosis (CF). This study aimed to better address the interplay between *P. aeruginosa* and chronically compromised airway environments through the examination of DPB *P. aeruginosa* strains, as existing literature regarding DPB is limited to case reports, case series, and clinical treatment guidelines. The evaluation of these features in the context of DPB, in tandem with prevailing knowledge of *P. aeruginosa* strains collected from more common chronic lung diseases (e.g., CF), can aid in the development of more effective strategies to combat respiratory *P. aeruginosa* infections in patients with chronic lung diseases.

## INTRODUCTION

Diffuse panbronchiolitis (DPB) is a rare, idiopathic, and severe obstructive pulmonary disease first recognized in Japan in the 1960s (1–3). Almost exclusively diagnosed in East Asian populations, DPB is characterized by bilateral lung disease with diffuse yellow nodules and inflammation of the respiratory bronchioles. Common symptoms include chronic sinusitis, cough, purulent sputum, breathlessness, wheezing, and weight loss (1–3). Although DPB is likely underdiagnosed worldwide due to its non-specific presentation, the mean age of diagnosis is 40 years old, corresponding with symptom onset (2). The cause of DPB is unclear; however, correlations between specific human leukocyte antigen (HLA) types and the development of DPB have been identified (1–10). HLA-B54, in Japanese populations, and HLA-A11, in Korean populations, are regarded as strong predictors of developing DPB (1–5). Additionally, polymorphisms in two novel mucin-like genes, termed panbronchiolitis-related mucin-like 1 and 2, are also associated with the onset of DPB (6, 7). Another study identified DPB-associated polymorphisms in *MUC5B*, a mucin gene, suggesting that alterations in mucins may contribute to pathogenesis (8). Other factors have been evaluated as well, such as lymphocyte activity (e.g., elevated CD8+ cell activity in the airway lumen of DPB patients), human beta-defensin regulation (e.g., antimicrobial peptides involved in innate immunity), and environmental cofactors (9, 10).

Following disease onset, symptom manifestations leave DPB patients vulnerable to bacterial infection within the lungs (1–3, 11–14). As a result of excess mucus production in the airway, DPB patients experience bronchiectasis and obstructive lung pathology (1–3, 8). This provides opportunistic bacteria, such as *Pseudomonas aeruginosa*, an ideal environment for colonization (1–3). *P. aeruginosa* is a Gram-negative bacterium known to infect individuals with immune-dysregulated conditions by utilizing a wide array of virulence mechanisms to evade immune recognition (15–20). Sputum cultures reveal the presence of *P. aeruginosa* in 22% of DPB patients, increasing in frequency to 60% after 4 years from disease onset. Furthermore, the 10-year survival rate of *P. aeruginosa*-infected patients is 12%, compared with 73% for uninfected patients (2, 3). Importantly, macrolide antibiotic therapies were used to treat chronic DPB lung infections at sub-bactericidal concentrations, which resulted in a nearly 30% improvement in DPB survival rates 5 years post-prognosis (21, 22). The success of macrolide therapy in DPB inspired the usage of these antibiotics to treat chronic lung infections in other conditions, such as cystic fibrosis (CF) and bronchiectasis (23, 24). DPB is a rare disease—11 cases per 100,000 persons were reported in Japan in the 1980s—but current disease rates are unknown due to limited epidemiologic surveillance (2). Access to *P. aeruginosa* strains from DPB patients is limited, and studies characterizing these *P. aeruginosa* strains in DPB have not yet been performed. Acknowledging both the susceptibility of DPB patients to *P. aeruginosa* infections and the correlation between *P. aeruginosa* infection and accelerated pulmonary decline in DPB, this study aimed to characterize 24 DPB *P. aeruginosa* strains for features known to enable chronic infection and impact the severity of other obstructive respiratory diseases, such as CF. Consequently, features such as swim/swarm motility, *in vitro* growth, lipid A structure, antibiotic susceptibility, genomic diversity, and O-antigen distribution were evaluated for each DPB *P. aeruginosa* strain.

## RESULTS

### Genetic distinctions exist between DPB, CF, and environmental *P. aeruginosa* strains

All DPB *P. aeruginosa* strains were initially subject to microbial identification via MALDI Biotyper analysis and confirmed as *P. aeruginosa* (Table S2). Following *P. aeruginosa* identification, phenotypic attributes of the DPB *P. aeruginosa* strains were observed. Differences in gross phenotypes (e.g., color, transparency, colony size, colony morphology, and mucoidy) were identified (Fig. S1).

**Fig 1:**
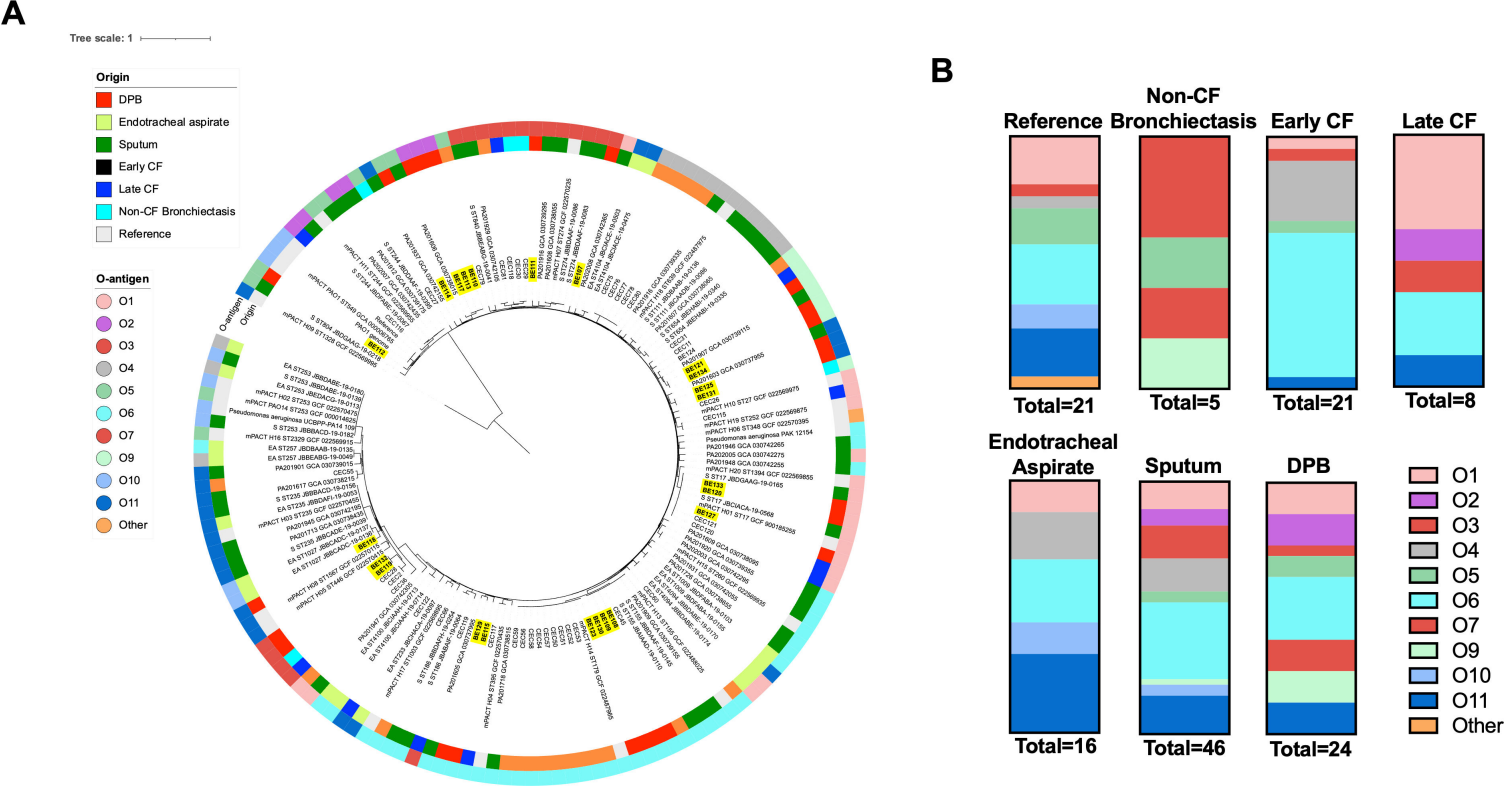
DPB *P. aeruginosa* strains are genomically distinct and have variable O-specific antigens. (**A**) Phylogenetic tree depicting the genetic relationships between the DPB *P. aeruginosa* strains and *P. aeruginosa* strains from diverse clinical and geographic origins. DPB strains are in bold and highlighted in yellow. Outside of the inferred phylogeny, each strain is shown in the inner ring as the clinical sample of the disease of origin, and the outer ring indicates the predicted O-antigen of each strain. Legends for each of these criteria are included. The phylogenies were inferred using the Northern Arizona SNP Pipeline(NASP) (30) and were visualized using the iTOL (v6.7.5) program (https://itol.embl.de/) (31). (**B**) O-antigen distribution of the DPB *P. aeruginosa* strains relative to reference, acute infection isolates, non-CF bronchiectasis, early CF, and late CF *P. aeruginosa* strains. Genomes of comparator *P. aeruginosa* strains were reported previously (25–28).

To evaluate DPB *P. aeruginosa* genomic variation, whole genome sequencing of each strain was conducted. Phylogenetic relationships between DPB, CF, non-CF bronchiectasis, and environmental *P. aeruginosa* strains were assessed via comparative genomics, as previously described (25). Genome assembly and comparative analysis of the CF, non-CF bronchiectasis, and regional and environmental *P*. aeruginosa strains were previously reported and are included as comparators (25–28). DPB *P. aeruginosa* strains do not form a single defined genomic clade when compared with strains obtained from chronic CF lung infections, acute infections, non-CF bronchiectasis, and environmental strains (Fig. 1A). There are some *P. aeruginosa* strains from DPB that do appear to exhibit clonal relationships with the following isolates being adjacent to each other in the phylogeny: BE110, BE113, and BE117; BE120, BE127, and BE133; BE125 and BE131; BE108, BE109, and BE130; BE121 and BE134; and BE119 and BE132 (Fig. 1A). These closely related isolates differ by on average 334 snps (SD 437 snp, range: 32–1111 snps), whereas the non-closely related strains differ on average by 24899 snps (SD 9427 snp, range: 6472–42862) snps). When the gene content of the *P. aeruginosa* strains from DPB was compared with that of *P. aeruginosa* strains from pulmonary isolations in Asia (Table S1), there were only 10 genes that were overrepresented among the DPB strains compared with other clinical isolates. The majority of these isolates are currently annotated as hypothetical, with the remainder being potentially surface-exposed (Table S3). Considering the genomic diversity of the examined strains, this lack of conserved DPB genes is not surprising. Although limited clinical information exists regarding *P. aeruginosa* collection from this DPB patient cohort, it is possible that these clonal DPB *P. aeruginosa* strains were isolated from the same patient, a pattern also observed in CF *P. aeruginosa* strains (29).

### Heterogeneity of DPB *P. aeruginosa* strain phenotype, motility, and *in vitro* growth

As motility plays an important role in *P. aeruginosa* virulence, flagellar swimming and type IV pili swarming motility were assessed in the DPB *P. aeruginosa* isolates. Variable motility was observed among the DPB *P. aeruginosa* samples, where some strains displayed relatively high motility function with average motility diameters over 15 mm, whereas others were non-motile (Table S4).

*In vitro* growth of the DPB *P. aeruginosa* strains was similarly diverse, ranging from fast to slow-growing strains. Bacterial growth curves were conducted to quantify differences in growth rate for each DPB *P. aeruginosa* strain grown in lysogeny broth (LB). The doubling times of the DPB *P. aeruginosa* samples spanned from 2.0 hours to 22.7 hours, as each strain displayed a unique growth ability (Fig. S2). The doubling time of PAO1 was 3.0 hours. No apparent relationship was observed between bacterial growth rate and other tested phenotypes (i.e., motility).

### Antibiotic susceptibility profiles of DPB *P. aeruginosa* strains are non-uniform

The antibiotic susceptibility profile of each DPB *P. aeruginosa* strain was identified using Kirby-Bauer disk diffusion assay (32). Twelve antibiotics were tested for each DPB *P. aeruginosa* strain, including first-line beta-lactams, aminoglycosides, macrolides, and polymyxins (Table 1). Similar to CF *P. aeruginosa* strains, the DPB *P. aeruginosa* strains varied in antibiotic susceptibility (Table 1) (17, 18, 33, 34). Uniform antibiotic susceptibility was only observed in response to polymyxin B and colistin (Table 1). Conversely, uniform antibiotic resistance was only observed in response to rifampicin and meropenem-vaborbactam (Table 1). Over 90% of the DPB *P. aeruginosa* strains were also resistant to ampicillin-sulbactam, trimethoprim, and erythromycin (Table 1). Susceptibility of the DPB *P. aeruginosa* strains ranged from as low as 25% (3/12) to as high as 58.3% (7/12) of all antibiotics tested.

**TABLE 1:** Antibiotic susceptibility data of *P. aeruginosa* DPB strains^*a*^^,^^*b*^

Antibiotic	BE107	BE108	BE109	BE110	BE111	BE112	BE113	BE114	BE115	BE117	BE118	BE119
Rifampicin (5 µg)	0	0	0	0	0	10	0	0	0	0	0	0
	R	R	R	R	R	R	R	R	R	R	R	R
Tetracycline (30 µg)	25	25	13	25	16	15	13	46	14	24	20	13
	S	S	I	S	S	S	I	S	I	S	S	I
Ampicillin/Sulbactam (20 µg)	15	0	0	0	0	12	0	0	0	0	0	0
	I	R	R	R	R	I	R	R	R	R	R	R
Polymyxin B (300 µg)	20	20	17	18	22	18	15	18	15	15	17	23
	S	S	S	S	S	S	S	S	S	S	S	S
Aztreonam (30 µg)	50	20	0	16	46	30	13	23	30	35	37	0
	S	I	R	I	S	S	R	S	S	S	S	R
Trimethoprim (5 µg)	0	0	0	0	0	12	0	0	0	0	0	0
	R	R	R	R	R	I	R	R	R	R	R	R
Tobramycin (10 µg)	26	26	20	0	33	27	0	35	15	0	23	34
	S	S	S	R	S	S	R	S	S	R	S	S
Erythromycin (15 µg)	0	0	0	0	0	28	0	25	0	0	0	0
	R	R	R	R	R	S	R	S	R	R	R	R
Meropenem (10 µg)	50	37	32	40	0	44	33	15	0	25	20	0
	S	S	S	S	R	S	S	I	R	S	S	R
Ceftazidime (30 µg)	20	13	0	13	0	10	0	0	0	0	0	0
	S	R	R	R	R	R	R	R	R	R	R	R
Meropenem/Vaborbactam (30 µg)	0	0	0	0	0	0	0	0	0	0	0	0
	R	R	R	R	R	R	R	R	R	R	R	R
Colistin (10 µg)	19	20	18	19	23	19	17	21	15	22	21	24
	S	S	S	S	S	S	S	S	S	S	S	S

Antibiotic	BE120	BE121	BE123	BE124	BE125	BE127	BE129	BE130	BE131	BE132	BE133	BE134
Rifampicin (5 µg)	0	0	0	16	0	0	0	0	0	0	0	0
	R	R	R	R	R	R	R	R	R	R	R	R
Tetracycline (30 µg)	20	12	20	12	0	11	17	0	0	13	11	0
	S	I	S	I	R	R	S	R	R	I	R	R
Ampicillin/Sulbactam (20 µg)	0	0	0	0	0	0	0	0	0	0	0	0
	R	R	R	R	R	R	R	R	R	R	R	R
Polymyxin B (300 µg)	22	12	20	20	15	16	15	17	15	25	20	25
	S	S	S	S	S	S	S	S	S	S	S	S
Aztreonam (30 µg)	30	40	45	45	20	19	35	35	25	50	35	42
	S	S	S	S	I	I	S	S	S	S	S	S
Trimethoprim (5 µg)	0	0	0	0	0	0	0	0	0	0	0	0
	R	R	R	R	R	R	R	R	R	R	R	R
Tobramycin (10 µg)	27	26	23	27	23	25	35	22	22	35	21	15
	S	S	S	S	S	S	S	S	S	S	S	S
Erythromycin (15 µg)	0	0	0	0	0	0	0	0	0	0	12	0
	R	R	R	R	R	R	R	R	R	R	R	R
Meropenem (10 µg)	13	15	22	34	0	16	22	30	17	0	43	52
	R	I	S	S	R	S	S	S	S	R	S	S
Ceftazidime (30 µg)	15	15	22	25	12	11	15	0	10	26	17	22
	I	I	S	S	R	R	I	R	R	S	I	S
Meropenem/Vaborbactam (30 µg)	0	0	0	0	0	0	0	0	0	0	0	0
	R	R	R	R	R	R	R	R	R	R	R	R
Colistin (10 µg)	19	18	21	19	16	17	17	19	16	24	20	19
	S	S	S	S	S	S	S	S	S	S	S	S

^*a*^ Tabulated summary of the Kirby-Bauer disk diffusion assay results for the DPB *P. aeruginosa* strains. Each numerical value represents the diameter of the zone of inhibition in millimeters. Diameter measures were utilized to define the DPB *P. aeruginosa* resistance profiles.

^*b*^ R = Resistant, I = Intermediate, S = Susceptible.

### O-antigen type and expression are variable in DPB *P. aeruginosa* strains

Differences in O-antigen type among DPB, CF, and environmental *P. aeruginosa* strains were additionally assessed via comparative genomics (using PAst) and serotyping (Fig. 1B) (35). The bioinformatic approach (i.e., PAst) assessed genetic components that encode O- specific antigen type, whereas the serologic approach allowed us to determine if O-antigen was expressed. This comprehensive approach was needed as many CF strains do not express full-length O-antigen (i.e., rough lipopolysaccharide; LPS), compared with acutely infectious strains that do express full-length O-antigen (i.e., smooth LPS) (36).

Although multiple O-antigen types were observed in our DPB cohort, the majority of DPB *P. aeruginosa* strains (6/24) were found to genetically encode the O6 type, a trend also observed among the 21 early CF *P. aeruginosa* strains (12/21) (Fig. 1B). The O6 serotype was also the most prevalent among the collections of clinical *P. aeruginosa* strains from respiratory sources in Japan (8/36) and China (9/28).(26, 27) The O1 type was the most prevalent among the late CF cohort (3/8) (Fig. 1B). DPB *P. aeruginosa* strains encode O2 or O16 and O7 or O8 O-antigen, which are not observed in our cohort of CF *P. aeruginosa* strains (Fig. 1B). In total, O1, O6, and O11 O-antigen types were the only types present in all of *P. aeruginosa* collection backgrounds examined (Fig. 1B).

For the DPB *P. aeruginosa* strains, LPS was isolated and O-antigen expression was determined using western immunoblot with three pools of O-antigen sera. Similar to *P. aeruginosa* obtained from CF chronic lung infection, a minority of DPB strains express O-antigen (5/24), suggesting the pressures that underlie a loss of O-antigen expression are similar between CF and DPB strains (Table S5).

### Gene-level bioinformatic approach to assess for genomic basis of strain variability

**Fig 2:**
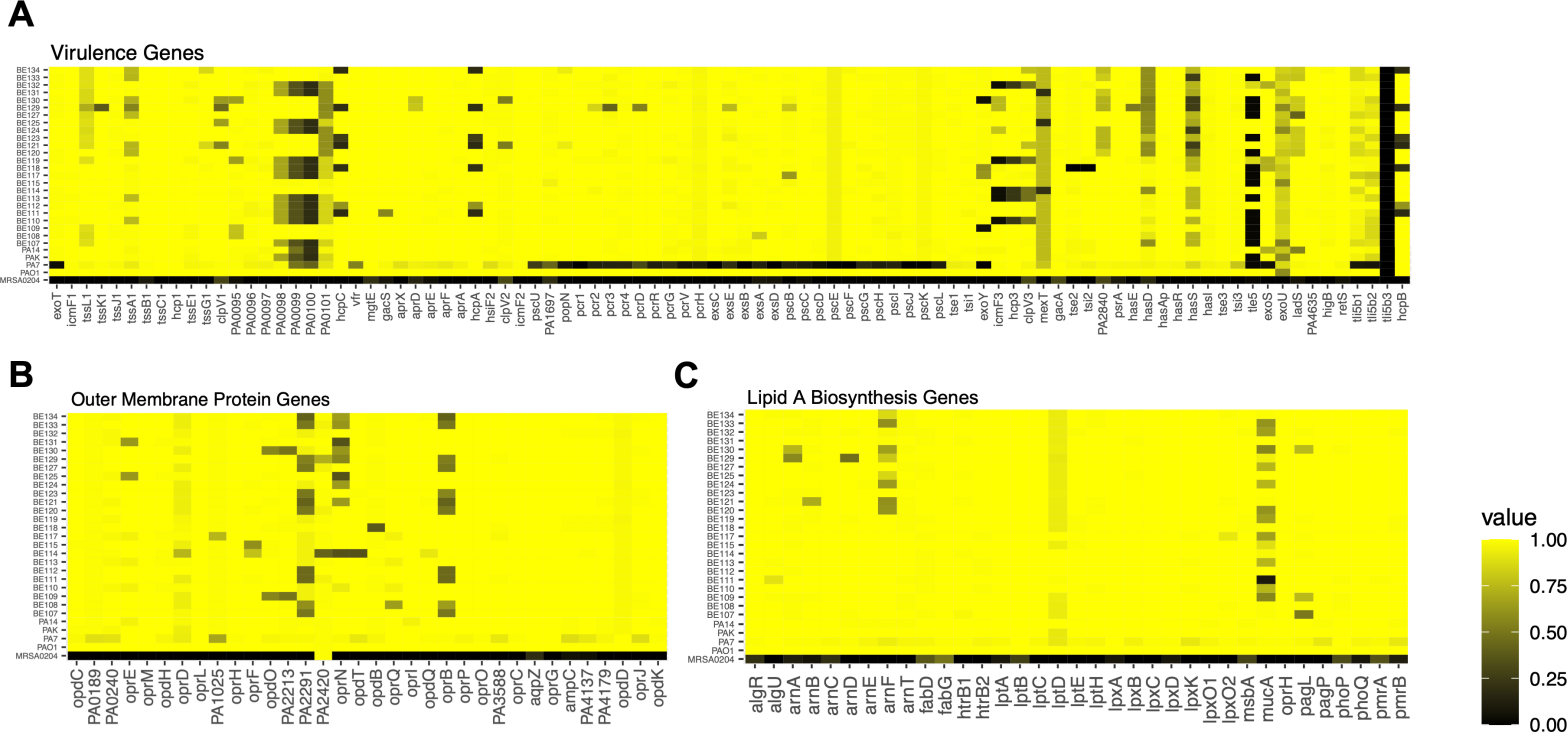
LS-BSR analysis of virulence and outer membrane protein genes. LS-BSR analysis was performed, where an LS-BSR score of 1 indicates 100% gene homology with reference sequence, and a score of 0 indicates no homology. *P. aeruginosa* reference gene sequences were obtained from a PAO1 consensus sequence (assembly accession GCF_000006765.1). Laboratory-adapted strains (PA14, PAK, PAO1, and PA7) and one *Staphylococcus aureus* strain was included in this analysis to serve as positive and negative controls, respectively. (**A**) Virulence genes were selected based on their role in *P. aeruginosa* pathogenesis (i.e., type-3 and type-6 secretion systems). (**B**) Outer membrane protein genes were selected based on their proposed role in bacterial survival and membrane homeostasis during infection. (**C**) Lipid A biosynthesis genes were selected based on their role in both lipid A synthesis and modification pathways.

We evaluated sequence divergence in other genes important for pathogenic success to better understand how DPB *P. aeruginosa* strains adapt to the human airway during chronic infection. We took a bioinformatic approach using a large-scale BLAST score ratio (LS-BSR) analysis. Notably, there are eight *P*. *aeruginosa* strains with sequence divergence in genes in the *arn* locus involved in 4-amino-L-arabinose addition to a terminal phosphate of lipid A of the glucosamine backbone (Fig. 2). These differences may explain the variability in polymyxin susceptibility, as 4-amino-L-arabinose modification of lipid A confers polymyxin resistance (37–39). Furthermore, sequence divergence in *mucA*, a negative regulator of alginate biosynthesis, was also observed, corroborating a mucoid phenotype (Fig. S1 and 2C).

When virulence genes were interrogated for sequence divergence, increased variability was observed, further confirming intra-strain heterogeneity. Interestingly, sequence diversity in *hcp* genes (*hcpA, hcpB,* and *hcpC*), involved in type-6 secretion system (T6SS) formation, was present in seven DPB strains (Fig. 2A). Sequence divergence in PA2840 (also known as *deaD*) was also observed for nine DPB strains. DeaD has RNA helicase activity that is important for the expression of type III secretion system (T3SS) genes involved in injecting bacterial effectors directly into host cells.

We also investigated outer membrane protein genes, which are crucial in understanding how *P. aeruginosa* interacts with its environment. Sequence divergence in *oprB* was observed in 11 strains (Fig. 2B). OprB is an outer membrane porin involved in glucose uptake, and its loss of function may suggest altered metabolism during chronic lung infection, a phenomenon proposed in *P. aeruginosa* chronic infection in CF (40)

### DPB *P. aeruginosa* strains demonstrate lipid A structural diversity

*P. aeruginosa* obtained from the airways of people with CF demonstrates an altered lipid A structure, which can induce variable signaling through innate immune pattern recognition receptors (25, 41–43). Modification of lipid A structure likely contributes to the ability of *P. aeruginosa* to chronically infect CF patients; therefore, we determined if lipid A structural diversity is also present in this cohort of DPB *P. aeruginosa* strains (41–43). Fast lipid analysis technique (FLAT) and matrix-assisted laser desorption/ionization time-of-flight (MALDI-TOF) mass spectrometry (MS) were used to evaluate lipid A structural modifications. LS-BSR was used to identify mutations in lipid A biosynthesis genes (Fig. 2C).

**Fig 3:**
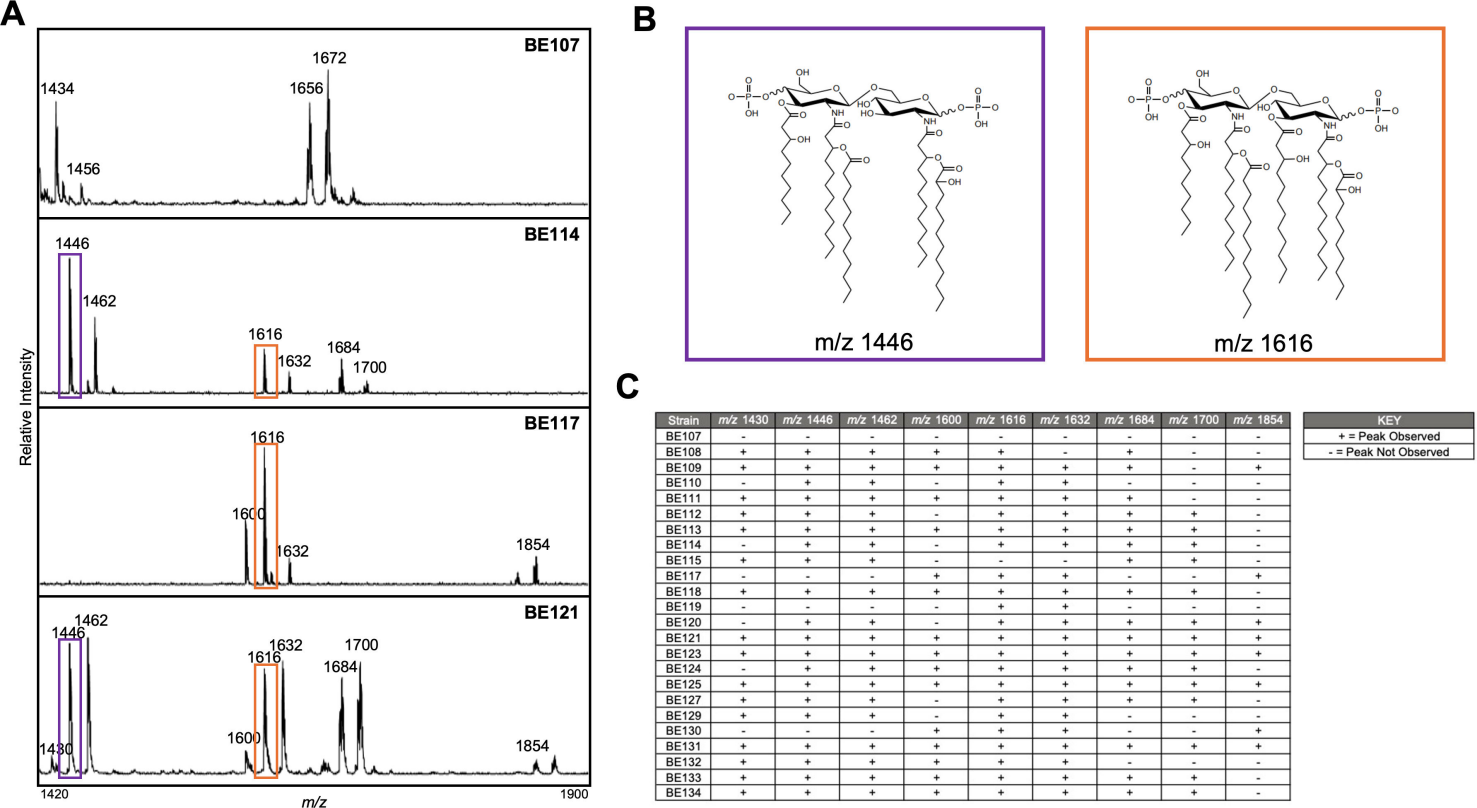
Lipid A structural variation present in *P. aeruginosa* strains obtained from subjects with diffuse panbronchiolitis. (**A**) Representative lipid A mass spectra of the DPB *P. aeruginosa* strain sample set. Canonical penta-acylated (*M/z* 1446) and hexa-acylated (*m/z* 1616) lipid A peaks are boxed in purple and orange, respectively. (**B**) Penta-acylated (*m/z* 1446, boxed in purple) and hexa-acylated (*m/z* 1616, boxed in orange) *P. aeruginosa* lipid A structures. (**C**) Tabulated summary of the DPB *P. aeruginosa* lipid A mass spectra documenting commonly observed *P. aeruginosa* peaks at *m/z* 1430, *m/z* 1446, *m/z* 1462, *m/z* 1600, *m/*z 1616, *m/z* 1632, *m/z* 1684, *m/z* 1700, and *m/z* 1854.

Using this MALDI-TOF MS approach, DPB *P. aeruginosa* strains demonstrated lipid A structural heterogeneity similar to that observed in *P. aeruginosa* strains from people with CF (Fig. 3A and C) (41–43). The associated lipid A mass spectra are provided in Fig. S3. Peaks at *m/z* 1446 and *m/z* 1616 represent penta-acylated and hexa-acylated lipid A structures, respectively, and are boxed in purple (*m/z* 1446) and orange (*m/z* 1616) (Fig. 3A and B) (41, 42). Peaks at *m/z* 1684 are a result of PagP acyltransferase activity, a lipid A phenotype often seen in CF *P. aeruginosa* strains, as the increase in mass reflects the addition of palmitate (C16, *m/z* ∆238) (Fig. 3A) (44). Also observed in CF *P. aeruginosa* strains, the absence of peaks at *m/z* 1430, *m/z* 1446, and *m/z* 1462 reflect a lack of PagL (lipid A 3-*O*-deacylase) activity (Fig. 3A and C) (45).

One of the DPB *P. aeruginosa* strains, BE107, exhibited a novel lipid A mass spectra peak configuration, with peaks observed at *m/z* 1656, *m/z* 1672, and *m/z* 1692 (Fig. 3A). Tandem mass spectrometry (MS/MS) and gas chromatography with flame-ionization detection (GC-FID) were used to further characterize this novel lipid A structure (Fig. S4). Possessing six acyl chains, the BE107 lipid A structure is distinct in its integration of a C16:1 cis-double bond about carbon 9 (Fig. S4C). To date, this lipid A structure has not been observed in any *P. aeruginosa* strain and highlights the structural plasticity of *P. aeruginosa* lipid A.

## DISCUSSION

Diffuse panbronchiolitis (DPB) is a severe chronic obstructive lung disease that can result in respiratory failure and fatality when untreated (2, 3). Patients with DPB present clinically with several non-specific respiratory symptoms and bronchiolectasis (1–3). Recognizing both the susceptibility of DPB patients to *P. aeruginosa* infections and the correlation between *P. aeruginosa* infection and advanced pulmonary decline, this study aimed to better understand features of DPB *P. aeruginosa* strains that both enable chronic infection and confer virulence impacting patient outcomes in both DPB and other obstructive respiratory diseases, such as CF (2, 3). Although many gaps exist in the knowledge of DPB pathophysiology, phenotypic and genotypic assessment of DPB *P. aeruginosa* strains can be a novel comparator to *P. aeruginosa* strains collected from other chronic lung diseases, such as CF.

Comparative genomics confirmed variation among DPB *P. aeruginosa* strains relative to each other and *P. aeruginosa* from other collection backgrounds (Fig. 1). The DPB *P. aeruginosa* strains appeared to group into several small regions on the phylogeny, indicating pockets of genetic similarity among strains in the DPB patient cohort (Fig. 1A). One of these clusters defines a clonal relationship between BE109 and BE130 (Fig. 1A), suggesting that the alternate strain identifications for BE109 and BE130 (KUD 003–1 and KUD 003–2, respectively; see Table S2) may indicate that these strains were collected from the same patient at different times. The O-antigen distribution of DPB *P. aeruginosa* strains was also variable (Fig. 1A). Furthermore, DPB *P. aeruginosa* strains displayed O-antigen diversity greater than the environmental and CF *P. aeruginosa* strains, supporting the hypothesis that DPB *P. aeruginosa* strains are phenotypically and genetically unique. Importantly, the majority of DPB *P. aeruginosa* strains express rough LPS (lacking full-length O-specific antigen), a characteristic once thought to be unique to CF *P. aeruginosa* strains (36, 46). This suggests that selective pressures on *P. aeruginosa* lipid A structure and O-antigen expression, once thought to be specific to the CF airway, are present in other conditions of chronic airway infection. One role of full-length O-antigen at the host-pathogen interface is to protect the bacterium from killing by complement (47); therefore, we speculate that the lack of complement-mediated killing in the small airways allows for selection of rough LPS *P. aeruginosa* mutants in both CF and DPB.

Upon initial characterization, colony morphology revealed several of these DPB strains to be mucoid (Fig. S1), and this mucoid phenotype is corroborated by sequence divergence in *mucA* (Fig. 2). Mucoidy, defined by constitutive alginate or other extracellular polysaccharide production, is associated with CF *P. aeruginosa* strains and not often seen in other disease contexts (48–50); therefore, these data suggest that mucoid may arise secondary to chronic lung infection, independent of cystic fibrosis transmembrane conductance regulator (CFTR) dysfunction. Swim and swarm motility assays were performed to assess the retention of motility machinery in the DPB *P. aeruginosa* strains, as CF *P. aeruginosa* strains can have altered motility during chronic infection (51). Haze and swarm diameters were variable among the DPB *P. aeruginosa* strains, indicating a non-uniform motility function (Table S4). Perhaps these differences are a result of the varying DPB patient airway immune responses, as the expressions of flagella, type IV pili, and other motility machinery are associated with enhanced virulence and immune recognition via pattern recognition receptors (15).

The lipid A structural diversity of DPB *P. aeruginosa* strains not only emphasizes their heterogeneity but also reveals that DPB *P. aeruginosa* strains modify lipid A similarly to CF *P. aeruginosa* strains. Lipid A structural heterogeneity is observed in CF *P. aeruginosa* strains obtained during chronic lung infection; however, here, we also reveal lipid A structural variation is present in DPB *P. aeruginosa* strains (41–43). Genetic variation in key lipid A biosynthesis genes, such as *htrB1*, *htrB2*, *pagL*, *pagP*, *lpxO1*, and *lpxO2*, support these findings (Fig. 2) (41–43, 45). These data suggest that each DPB *P. aeruginosa* strain retains a specific lipid A structure, which may improve bacterial barrier function and immune evasion specific to its DPB patient of origin, ultimately enabling sustained chronic infection as observed in CF *P. aeruginosa* strains (41–43, 45).

Additional experimentation (MS/MS, GC-FID) elucidated the novel lipid A peak configuration displayed for BE107 (Fig. 3A; Fig. S4). The BE107 lipid A structure is unique, with its integration of a C16:1 cis-double bond about carbon 9 (Fig. S4C). This cis-double bond creates a kink in the C16 acyl chain, as the adjacent single bonds point in the same direction. Regarding three-dimensional orientation, we hypothesize that this kink induces spatial separation of the surrounding LPS molecules (41, 43, 52). Acknowledging that BE107 was one of the least resistant strains to the antibiotic panel tested, the supposed spacing of the outer membrane induced by the C16:1 cis-double bond kink may correlate with these findings, as antibiotics would theoretically confront less physical opposition when entering the bacteria (Table 1). Further experimentation identifying which lipid A modifying enzymes are involved in adding a C16:1 cis-double bond may be informative for future experimentation, as this acyl chain structure is not seen in canonical *P. aeruginosa* lipid A.

Variable antibiotic susceptibility profiles were revealed for the DPB *P. aeruginosa* strains (Table 1). Across the antibiotic panel tested, no DPB *P. aeruginosa* strains displayed identical susceptibility. Resistance to erythromycin, the main therapeutic agent used to treat DPB patients, was observed in roughly 91% of the DPB *P. aeruginosa* strains, suggesting that treatment of chronic *P. aeruginosa* infections can lead to antibiotic resistance, especially after months or years of infection and subsequent antibiotic exposure (Table 1). Noting that inhaled colistin has been used as a therapeutic approach to combat bacterial infection in CF patients, a similar approach may be promising to treat DPB patients with refractory *P. aeruginosa* infections as the DPB *P. aeruginosa* strains display uniform susceptibility to colistin (53).

The results of this study show that our cohort of 24 DPB *P. aeruginosa* strains exhibit considerable phenotypic and genotypic diversity, with similarities to both acute and chronic *P. aeruginosa* infections. This diversity of colonization sources underscores the challenges in treating *P. aeruginosa* infections in DPB, CF, and other obstructive respiratory diseases. Similarities observed between DPB and CF *P. aeruginosa* strains prove informative for future studies, as this work heightens the current understanding of the interplay between *P. aeruginosa* and chronic lung disease.

## MATERIALS AND METHODS

### Bacterial strains

Archived *P. aeruginosa* strains were obtained from the airways of subjects with diffuse panbronchiolitis (before the year 2000)—strain details are outlined in Table S1 (Dr. Samuel M. Moskowitz, University of Washington; currently Design Therapeutics). Strains were stored at −80°C in 25% glycerol. Strains were streaked on lysogenic broth (LB) agar utilizing a tertiary streak pattern and incubated at 37°C for 18 hours with inversion. Liquid cultures were inoculated utilizing single colonies and incubated at 37°C for 18 hours, shaking at 180 rpm. Due to the archived nature of these historic samples, no associated clinical data exist for this cohort of *P. aeruginosa* strains. Genomic sequences of other *P. aeruginosa* strains used in this study (from environmental, regional, and CF sources) are described previously (25–28, 54). Metadata for strains are included in Tables S1a and b, including GenBank accession numbers.

### Fast lipid analysis technique

Lipid A structural analysis was performed, as previously described (55). A single colony was scraped onto a steel target plate in duplicate with a toothpick. FLAT extraction buffer (1 µL; 0.2 M anhydrous citric acid, 0.1 M trisodium citrate dihydrate) was pipetted over bacterial spots. The target plate was incubated at 100°C for 30 minutes, washed with ddH_2_O and air-dried. Mass spectra were collected in negative-ion mode using a Bruker MicroFlex®. Mass spectra were analyzed using FlexAnalysis v3.4 software.

### Whole-genome sequencing

The genomes of all strains analyzed in this study were sequenced, as previously described (56). Sample libraries were prepared with the Illumina DNA prep kit (cat. num. 20018705) and sequenced on the Illumina NextSeq 2500, producing 2  ×  151 bp paired-end reads. All software was used with default values. Raw sequencing reads were filtered to remove contaminating phiX reads using BBDuk, one of the BBTools software suites (sourceforge.net/projects/bbmap/). The raw reads were also filtered to remove contaminating Illumina adaptor sequences and quality-trimmed using Trimmomatic v. 0.36 (57). The resulting filtered reads were assembled using SPAdes v. 3.13.0 (58).

### Phylogenomic analysis

Comparison was conducted using an *in silico* genotype (ISG) (59). Single nucleotide polymorphisms (SNPs) were detected relative to the reference *P. aeruginosa* strain PAO1 (GenBank accession #NC_002516.2) using the ISG that uses the NUCmer (v.3.22) program (60) for SNP detection. SNP sites that were identified in all analyzed genomes were concatenated and used to construct a maximum likelihood phylogeny using RAxML (v7.2.8) (61). All phylogenies were visualized using the iTOL (v6.7.5) program (https://itol.embl.de/) (61).

SNP distances were determined using snp-dists with default values (https://github.com/tseemann/snp-dists; Data set 1).

### Genome-based comparisons

Roary/Scoary: All genomes used in subsequent analyses (Table S1a/S1b) were annotated via Prodigal (62) to generate *de novo* CDS and then PROKKA v1.14.6 (63). The resulting general feature formats (GFF) were analyzed to identify core and accessory genes using Roary v3.13.0 (64) and Scoary version 1.6.16 (65).

### *P. aeruginosa* O-antigen sequence type determination

O-antigen types were identified using *P. aeruginosa* serotyper (PAst) program, as previously described (35).

### LPS purification and visualization

LPS was purified as previously described (66) with slight modifications. Briefly, *P. aeruginosa* overnight cultures grown on LB agar and were resuspended in PBS and normalized to OD_600_ = 0.5 (~1e9 CFU / mL). Washed bacteria were then pelleted by centrifugation and resuspended in 200 µL sodium dodecyl sulfate (SDS) sample buffer (BioRad) and briefly boiled. Samples were incubated with 2.5 mL DNaseI (10 mg/mL), and 2.5 mL RNase (10 µg/mL) for 30 minutes at 37°C, followed by 3 hours of incubation with 10 mL Proteinase K (10 mg/mL) at 59°C. Ten microliters of purified LPS were loaded and separated on a 12% Tris-glycine gel (BioRad) using SDS-PAGE and then visualized by western blotting. Samples were probed with *P. aeruginosa* serogroup rabbit polyvalent antibodies (Denka Seiken, Tokyo Japan) followed by incubation with anti-rabbit secondary antibodies conjugated to horseradish peroxidase (HRP) (Sigma). Reactions were visualized by the addition of Clarity Max Western ECL Substrate (BioRad).

### Large-scale BLAST score ratio analysis

Genomes of DPB *P. aeruginosa* strains were compared using LS-BSR analysis, as previously described (67). Gene sets were chosen to investigate the genetic variation of DPB *P. aeruginosa* strains and differences in bacterial functions, such as virulence or membrane maintenance. Gene sequences were obtained using the reference *P. aeruginosa* strain PAO1 (GenBank accession no. NC_002516.2). Heat maps of the LS-BSR values were generated in RStudio using ggplot2.

## SUPPLEMENTAL MATERIAL

Figure S1 spectrum.00530-24-s0001.pdf Photographs of the DPB *P. aeruginosa* strains utilized in this study.

Figure S2 spectrum.00530-24-s0002.docx In vitro growth curves.

Figure S3 spectrum.00530-24-s0003.docx Lipid A mass spectra of each DPB *P. aeruginosa* strain in the sample set (BE107-BE134).

Figure S4 spectrum.00530-24-s0004.docx Structural analysis of BE107.

Table S1 spectrum.00530-24-s0005.xlsx Strains used in this study.

Supplemental tables spectrum.00530-24-s0006.docx Tables S2 to S5.

Supplemental materials spectrum.00530-24-s0007.docx Supplemental methods.

ASM does not own the copyrights to Supplemental Material that may be linked to, or accessed through, an article. The authors have granted ASM a non-exclusive, world-wide license to publish the Supplemental Material files. Please contact the corresponding author directly for reuse.

## References

[1] Iwata M, Colby TV, Kitaichi M. 1994. Diffuse panbronchiolitis: diagnosis and distinction from various pulmonary diseases with centrilobular interstitial foam cell accumulations. Hum Pathol 25:357–363. doi:10.1016/0046-8177(94)90143-0 8163268

[2] Poletti V, Casoni G, Chilosi M, Zompatori M. 2006. Diffuse panbronchiolitis. Eur Respir J 28:862–871. doi:10.1183/09031936.06.00131805 17012632

[3] Azuma A, Kudoh S. 2006. Diffuse panbronchiolitis in East Asia. Respirology 11:249–261. doi:10.1111/j.1440-1843.2006.00845.x 16635082

[4] Sugiyama Y, Kudoh S, Maeda H, Suzaki H, Takaku F. 1990. Analysis of HLA antigens in patients with diffuse panbronchiolitis. Am Rev Respir Dis 141:1459–1462. doi:10.1164/ajrccm/141.6.1459 2350086

[5] Park MH, Kim YW, Yoon HI, Yoo CG, Han SK, Shim YS, Kim WD. 1999. Association of HLA class I antigens with diffuse panbronchiolitis in Korean patients. Am J Respir Crit Care Med 159:526–529. doi:10.1164/ajrccm.159.2.9805047 9927368

[6] Hijikata M, Matsushita I, Tanaka G, Tsuchiya T, Ito H, Tokunaga K, Ohashi J, Homma S, Kobashi Y, Taguchi Y, Azuma A, Kudoh S, Keicho N. 2011. Molecular cloning of two novel mucin-like genes in the disease-susceptibility locus for diffuse panbronchiolitis. Hum Genet 129:117–128. doi:10.1007/s00439-010-0906-4 20981447

[7] Keicho N, Ohashi J, Tamiya G, Nakata K, Taguchi Y, Azuma A, Ohishi N, Emi M, Park MH, Inoko H, Tokunaga K, Kudoh S. 2000. Fine localization of a major disease-susceptibility locus for diffuse panbronchiolitis. Am J Hum Genet 66:501–507. doi:10.1086/302786 10677310 PMC1288103

[8] Kamio K, Matsushita I, Hijikata M, Kobashi Y, Tanaka G, Nakata K, Ishida T, Tokunaga K, Taguchi Y, Homma S, Nakata K, Azuma A, Kudoh S, Keicho N. 2005. Promoter analysis and aberrant expression of the MUC5B gene in diffuse panbronchiolitis. Am J Respir Crit Care Med 171:949–957. doi:10.1164/rccm.200409-1168OC 15709052

[9] Mukae H, Kadota J, Kohno S, Kusano S, Morikawa T, Matsukura S, Hara K. 1995. Increase in activated CD8+ cells in bronchoalveolar lavage fluid in patients with diffuse panbronchiolitis. Am J Respir Crit Care Med 152:613–618. doi:10.1164/ajrccm.152.2.7633715 7633715

[10] Hiratsuka T, Mukae H, Iiboshi H, Ashitani J, Nabeshima K, Minematsu T, Chino N, Ihi T, Kohno S, Nakazato M. 2003. Increased concentrations of human beta-defensins in plasma and bronchoalveolar lavage fluid of patients with diffuse panbronchiolitis. Thorax 58:425–430. doi:10.1136/thorax.58.5.425 12728165 PMC1746672

[11] Lin X, Lu J, Yang M, Dong BR, Wu HM. 2015. Macrolides for diffuse panbronchiolitis. Cochrane Database Syst Rev 1:CD007716. doi:10.1002/14651858.CD007716.pub4 25618845 PMC6464977

[12] Trédaniel J, Cazals-Hatem D, Zalcman G, d’Agay MF, Capron F. 1994. Diffuse panbronchiolitis: efficacy of low-dose erythromycin. Respir Med 88:479–480. doi:10.1016/s0954-6111(05)80057-5 7938804

[13] Kudoh S, Azuma A, Yamamoto M, Izumi T, Ando M. 1998. Improvement of survival in patients with diffuse panbronchiolitis treated with low-dose erythromycin. Am J Respir Crit Care Med 157:1829–1832. doi:10.1164/ajrccm.157.6.9710075 9620913

[14] Friedlander AL, Albert RK. 2010. Chronic macrolide therapy in inflammatory airways diseases. Chest 138:1202–1212. doi:10.1378/chest.10-0196 21051396

[15] Moradali MF, Ghods S, Rehm BHA. 2017. Pseudomonas aeruginosa lifestyle: a paradigm for adaptation, survival, and persistence. Front Cell Infect Microbiol 7:39. doi:10.3389/fcimb.2017.00039 28261568 PMC5310132

[16] Malhotra S, Hayes D, Wozniak DJ. 2019. Cystic fibrosis and Pseudomonas aeruginosa: the host-microbe interface. Clin Microbiol Rev 32:e00138-18. doi:10.1128/CMR.00138-18 31142499 PMC6589863

[17] Davies JC. 2002. Pseudomonas aeruginosa in cystic fibrosis: pathogenesis and persistence. Paediatr Respir Rev 3:128–134. doi:10.1016/s1526-0550(02)00003-3 12297059

[18] Rossi E, La Rosa R, Bartell JA, Marvig RL, Haagensen JAJ, Sommer LM, Molin S, Johansen HK. 2021. Pseudomonas aeruginosa adaptation and evolution in patients with cystic fibrosis. Nat Rev Microbiol 19:331–342. doi:10.1038/s41579-020-00477-5 33214718

[19] Hogardt M, Heesemann J. 2013 Microevolution of Pseudomonas aeruginosa to a chronic pathogen of the cystic fibrosis lung. Curr Top Microbiol Immunol 358:91–118. doi:10.1007/82_2011_199 22311171

[20] Stefani S, Campana S, Cariani L, Carnovale V, Colombo C, Lleo MM, Iula VD, Minicucci L, Morelli P, Pizzamiglio G, Taccetti G. 2017. Relevance of multidrug-resistant Pseudomonas aeruginosa infections in cystic fibrosis. Int J Med Microbiol 307:353–362. doi:10.1016/j.ijmm.2017.07.004 28754426

[21] Kricker JA, Page CP, Gardarsson FR, Baldursson O, Gudjonsson T, Parnham MJ. 2021. Nonantimicrobial actions of macrolides: overview and perspectives for future development. Pharmacol Rev 73:233–262. doi:10.1124/pharmrev.121.000300 34716226

[22] Shimizu T, Suzaki H. 2016. Past, present and future of macrolide therapy for chronic rhinosinusitis in Japan. Auris Nasus Larynx 43:131–136. doi:10.1016/j.anl.2015.08.014 26441370

[23] Høiby N. 1994. Diffuse panbronchiolitis and cystic fibrosis: East meets West. Thorax 49:531–532. doi:10.1136/thx.49.6.531 8016786 PMC474936

[24] Sun J, Li Y. 2022. Long-term, low-dose macrolide antibiotic treatment in pediatric chronic airway diseases. Pediatr Res 91:1036–1042. doi:10.1038/s41390-021-01613-4 34120139 PMC9122820

[25] Hofstaedter CE, Chandler CE, Met CM, Gillespie JJ, Harro JM, Goodlett DR, Rasko DA, Ernst RK. 2024. Divergent Pseudomonas aeruginosa LpxO enzymes perform site-specific lipid A 2-hydroxylation. mBio 15:e0282323. doi:10.1128/mbio.02823-23 38131669 PMC10865791

[26] Hu Z, Zhou L, Tao X, Li P, Zheng X, Zhang W, Tan Z. 2024. Antimicrobial resistance survey and whole-genome analysis of nosocomial P. aeruginosa isolated from eastern province of China in 2016–2021. Ann Clin Microbiol Antimicrob 23. doi:10.1186/s12941-023-00656-1 PMC10858563 38336730

[27] Yano H, Hayashi W, Kawakami S, Aoki S, Anzai E, Zuo H, Kitamura N, Hirabayashi A, Kajihara T, Kayama S, Sugawara Y, Yahara K, Sugai M. 2024. Nationwide genome surveillance of carbapenem-resistant Pseudomonas aeruginosa in Japan. Antimicrob Agents Chemother 68:e0166923. doi:10.1128/aac.01669-23 38564665 PMC11064530

[28] Tueffers L, Batra A, Zimmermann J, Botelho J, Buchholz F, Liao J, Mendoza Mejía N, Munder A, Klockgether J, Tüemmler B, Rupp J, Schulenburg H. 2024. Variation in the response to antibiotics and life-history across the major Pseudomonas aeruginosa clone type (mPact) panel. Microbiol Spectr 12:e0014324. doi:10.1128/spectrum.00143-24 38860784 PMC11218531

[29] Marvig RL, Sommer LM, Molin S, Johansen HK. 2015. Convergent evolution and adaptation of Pseudomonas aeruginosa within patients with cystic fibrosis. Nat Genet 47:57–64. doi:10.1038/ng.3148 25401299

[30] Sahl JW, Lemmer D, Travis J, Schupp JM, Gillece JD, Aziz M, Driebe EM, Drees KP, Hicks ND, Williamson CHD, Hepp CM, Smith DE, Roe C, Engelthaler DM, Wagner DM, Keim P. 2016. NASP: an accurate, rapid method for the identification of SNPs in WGS datasets that supports flexible input and output formats. Microb Genom 2:e000074. doi:10.1099/mgen.0.000074 28348869 PMC5320593

[31] Letunic I, Bork P. 2021. Interactive Tree Of Life (iTOL) v5: an online tool for phylogenetic tree display and annotation. Nucleic Acids Res 49:W293–W296. doi:10.1093/nar/gkab301 33885785 PMC8265157

[32] Jorgensen JH, Turnidge JD. 2015. Chapter 71, Susceptibility test methods: dilution and disk diffusion methods, p 1253–1273. In Manual of clinical microbiology. ASM Press.

[33] Rees VE, Deveson Lucas DS, López-Causapé C, Huang Y, Kotsimbos T, Bulitta JB, Rees MC, Barugahare A, Peleg AY, Nation RL, Oliver A, Boyce JD, Landersdorfer CB. 2019. Characterization of hypermutator Pseudomonas aeruginosa isolates from patients with cystic fibrosis in Australia. Antimicrob Agents Chemother 63:e02538-18. doi:10.1128/AAC.02538-18 30745381 PMC6437500

[34] Zhao K, Yuan Y, Li J, Pan W, Yan C, Fu H, Lin J, Yue B, Wang X, Gou X, Chu Y, Zhou Y. 2019. Phenotypic and genetic characterization of Pseudomonas aeruginosa isolate COP2 from the lungs of COPD patients in China. Pathog Dis 77:ftz038. doi:10.1093/femspd/ftz038 31348491

[35] Thrane SW, Taylor VL, Lund O, Lam JS, Jelsbak L. 2016. Application of whole-genome sequencing data for O-specific antigen analysis and in silico serotyping of Pseudomonas aeruginosa isolates. J Clin Microbiol 54:1782–1788. doi:10.1128/JCM.00349-16 27098958 PMC4922119

[36] Hancock RE, Mutharia LM, Chan L, Darveau RP, Speert DP, Pier GB. 1983. Pseudomonas aeruginosa isolates from patients with cystic fibrosis: a class of serum-sensitive, nontypable strains deficient in lipopolysaccharide O side chains. Infect Immun 42:170–177. doi:10.1128/iai.42.1.170-177.1983 6413410 PMC264539

[37] Moskowitz SM, Ernst RK, Miller SI. 2004. PmrAB, A two-component regulatory system of Pseudomonas aeruginosa that modulates resistance to cationic antimicrobial peptides and addition of aminoarabinose to lipid A. J Bacteriol 186:575–579. doi:10.1128/JB.186.2.575-579.2004 14702327 PMC305751

[38] Boll M, Radziejewska-Lebrecht J, Warth C, Krajewska-Pietrasik D, Mayer H. 1994. 4-Amino-4-deoxy-l-arabinose in LPS of enterobacterial R-mutants and its possible role for their polymyxin reactivity. FEMS Immunol Med Microbiol 8:329–341. doi:10.1111/j.1574-695X.1994.tb00460.x 8061656

[39] Olaitan AO, Morand S, Rolain JM. 2014. Mechanisms of polymyxin resistance: acquired and intrinsic resistance in bacteria. Front Microbiol 5:643. doi:10.3389/fmicb.2014.00643 25505462 PMC4244539

[40] La Rosa R, Johansen HK, Molin S. 2019. Adapting to the airways: metabolic requirements of Pseudomonas aeruginosa during the infection of cystic fibrosis patients. Metabolites 9:234. doi:10.3390/metabo9100234 31623245 PMC6835255

[41] Buchanan PJ, Ernst RK, Elborn JS, Schock B. 2009. Role of CFTR, Pseudomonas aeruginosa and Toll-like receptors in cystic fibrosis lung inflammation. Biochem Soc Trans 37:863–867. doi:10.1042/BST0370863 19614608

[42] Ernst RK, Moskowitz SM, Emerson JC, Kraig GM, Adams KN, Harvey MD, Ramsey B, Speert DP, Burns JL, Miller SI. 2007. Unique lipid A modifications in Pseudomonas aeruginosa isolated from the airways of patients with cystic fibrosis. J Infect Dis 196:1088–1092. doi:10.1086/521367 17763333 PMC2723782

[43] Park BS, Lee JO. 2013. Recognition of lipopolysaccharide pattern by TLR4 complexes. Exp Mol Med 45:e66. doi:10.1038/emm.2013.97 24310172 PMC3880462

[44] Thaipisuttikul I, Hittle LE, Chandra R, Zangari D, Dixon CL, Garrett TA, Rasko DA, Dasgupta N, Moskowitz SM, Malmström L, Goodlett DR, Miller SI, Bishop RE, Ernst RK. 2014. A divergent Pseudomonas aeruginosa palmitoyltransferase essential for cystic fibrosis-specific lipid A. Mol Microbiol 91:158–174. doi:10.1111/mmi.12451 24283944 PMC3935289

[45] Ernst RK, Adams KN, Moskowitz SM, Kraig GM, Kawasaki K, Stead CM, Trent MS, Miller SI. 2006. The Pseudomonas aeruginosa lipid A deacylase: selection for expression and loss within the cystic fibrosis airway. J Bacteriol 188:191–201. doi:10.1128/JB.188.1.191-201.2006 16352835 PMC1317579

[46] Lam MY, McGroarty EJ, Kropinski AM, MacDonald LA, Pedersen SS, Høiby N, Lam JS. 1989. Occurrence of a common lipopolysaccharide antigen in standard and clinical strains of Pseudomonas aeruginosa. J Clin Microbiol 27:962–967. doi:10.1128/jcm.27.5.962-967.1989 2501356 PMC267463

[47] Hastings CJ, Syed SS, Marques CNH. 2023. Subversion of the complement system by Pseudomonas aeruginosa. J Bacteriol 205:e0001823. doi:10.1128/jb.00018-23 37436150 PMC10464199

[48] Malhotra S, Hayes D, Wozniak DJ. 2019. Mucoid Pseudomonas aeruginosa and regional inflammation in the cystic fibrosis lung. J Cyst Fibros 18:796–803. doi:10.1016/j.jcf.2019.04.009 31036488 PMC6815243

[49] Li Z, Kosorok MR, Farrell PM, Laxova A, West SEH, Green CG, Collins J, Rock MJ, Splaingard ML. 2005. Longitudinal development of mucoid Pseudomonas aeruginosa infection and lung disease progression in children with cystic fibrosis. JAMA 293:581–588. doi:10.1001/jama.293.5.581 15687313

[50] Boucher JC, Yu H, Mudd MH, Deretic V. 1997. Mucoid Pseudomonas aeruginosa in cystic fibrosis: characterization of muc mutations in clinical isolates and analysis of clearance in a mouse model of respiratory infection. Infect Immun 65:3838–3846. doi:10.1128/iai.65.9.3838-3846.1997 9284161 PMC175548

[51] Winstanley C, O’Brien S, Brockhurst MA. 2016. Pseudomonas aeruginosa evolutionary adaptation and diversification in cystic fibrosis chronic lung infections. Trends Microbiol 24:327–337. doi:10.1016/j.tim.2016.01.008 26946977 PMC4854172

[52] Rocchetta HL, Burrows LL, Lam JS. 1999. Genetics of O-antigen biosynthesis in Pseudomonas aeruginosa. Microbiol Mol Biol Rev 63:523–553. doi:10.1128/MMBR.63.3.523-553.1999 10477307 PMC103745

[53] Bruguera-Avila N, Marin A, Garcia-Olive I, Radua J, Prat C, Gil M, Ruiz-Manzano J. 2017. Effectiveness of treatment with nebulized colistin in patients with COPD. Int J Chron Obstruct Pulmon Dis 12:2909–2915. doi:10.2147/COPD.S138428 29042767 PMC5634377

[54] Chandler CE, Hofstaedter CE, Hazen TH, Rasko DA, Ernst RK. 2023. Genomic and functional characterization of longitudinal Pseudomonas aeruginosa isolates from young patients with cystic fibrosis. Microbiol Spectr 11:e0155623. doi:10.1128/spectrum.01556-23 37358436 PMC10433850

[55] Sorensen M, Chandler CE, Gardner FM, Ramadan S, Khot PD, Leung LM, Farrance CE, Goodlett DR, Ernst RK, Nilsson E. 2020. Rapid microbial identification and colistin resistance detection via MALDI-TOF MS using a novel on-target extraction of membrane lipids. Sci Rep 10:21536. doi:10.1038/s41598-020-78401-3 33299017 PMC7725828

[56] Chandler CE, Horspool AM, Hill PJ, Wozniak DJ, Schertzer JW, Rasko DA, Ernst RK. 2019. Genomic and phenotypic diversity among ten laboratory isolates of Pseudomonas aeruginosa PAO1. J Bacteriol 201:e00595-18. doi:10.1128/JB.00595-18 30530517 PMC6379574

[57] Bolger AM, Lohse M, Usadel B. 2014. Trimmomatic: a flexible trimmer for Illumina sequence data. Bioinformatics 30:2114–2120. doi:10.1093/bioinformatics/btu170 24695404 PMC4103590

[58] Nurk S, Bankevich A, Antipov D, Gurevich AA, Korobeynikov A, Lapidus A, Prjibelski AD, Pyshkin A, Sirotkin A, Sirotkin Y, Stepanauskas R, Clingenpeel SR, Woyke T, McLean JS, Lasken R, Tesler G, Alekseyev MA, Pevzner PA. 2013. Assembling single-cell genomes and mini-metagenomes from chimeric MDA products. J Comput Biol 20:714–737. doi:10.1089/cmb.2013.0084 24093227 PMC3791033

[59] Hazen TH, Kaper JB, Nataro JP, Rasko DA. 2015. Comparative genomics provides insight into the diversity of the attaching and effacing Escherichia coli virulence plasmids. Infect Immun 83:4103–4117. doi:10.1128/IAI.00769-15 26238712 PMC4567640

[60] Delcher AL, Salzberg SL, Phillippy AM. 2003. Using MUMmer to identify similar regions in large sequence sets. Curr Protoc Bioinformatics:Unit. doi:10.1002/0471250953.bi1003s00 18428693

[61] Stamatakis A. 2006. RAxML-VI-HPC: maximum likelihood-based phylogenetic analyses with thousands of taxa and mixed models. Bioinformatics 22:2688–2690. doi:10.1093/bioinformatics/btl446 16928733

[62] Hyatt D, Chen GL, Locascio PF, Land ML, Larimer FW, Hauser LJ. 2010. Prodigal: prokaryotic gene recognition and translation initiation site identification. BMC Bioinformatics 11:119. doi:10.1186/1471-2105-11-119 20211023 PMC2848648

[63] Seemann T. 2014. Prokka: rapid prokaryotic genome annotation. Bioinformatics 30:2068–2069. doi:10.1093/bioinformatics/btu153 24642063

[64] Page AJ, Cummins CA, Hunt M, Wong VK, Reuter S, Holden MTG, Fookes M, Falush D, Keane JA, Parkhill J. 2015. Roary: rapid large-scale prokaryote pan genome analysis. Bioinformatics 31:3691–3693. doi:10.1093/bioinformatics/btv421 26198102 PMC4817141

[65] Brynildsrud O, Bohlin J, Scheffer L, Eldholm V. 2016. Rapid scoring of genes in microbial pan-genome-wide association studies with Scoary. Genome Biol 17:238. doi:10.1186/s13059-016-1108-8 27887642 PMC5124306

[66] Moustafa DA, DiGiandomenico A, Raghuram V, Schulman M, Scarff JM, Davis MR, Varga JJ, Dean CR, Goldberg JB. 2023. Efficacy of a Pseudomonas aeruginosa serogroup O9 vaccine. Infect Immun 91:e0024723. doi:10.1128/iai.00247-23 37991349 PMC10715167

[67] Sahl JW, Caporaso JG, Rasko DA, Keim P. 2014. The large-scale blast score ratio (LS-BSR) pipeline: a method to rapidly compare genetic content between bacterial genomes. PeerJ 2:e332. doi:10.7717/peerj.332 24749011 PMC3976120

