# Supplementary material for: Characterization of Pseudomonas aeruginosa from subjects with diffuse panbronchiolitis
Source: Microbiol Spectr. 2024 Oct 8;12(11):e00530-24. doi: 10.1128/spectrum.00530-24 (PMC11537112; doi:10.1128/spectrum.00530-24)
Supplement: Figure S1 — Photographs of the DPB P. aeruginosa strains utilized in this study. [file spectrum.00530-24-s0001.pdf]

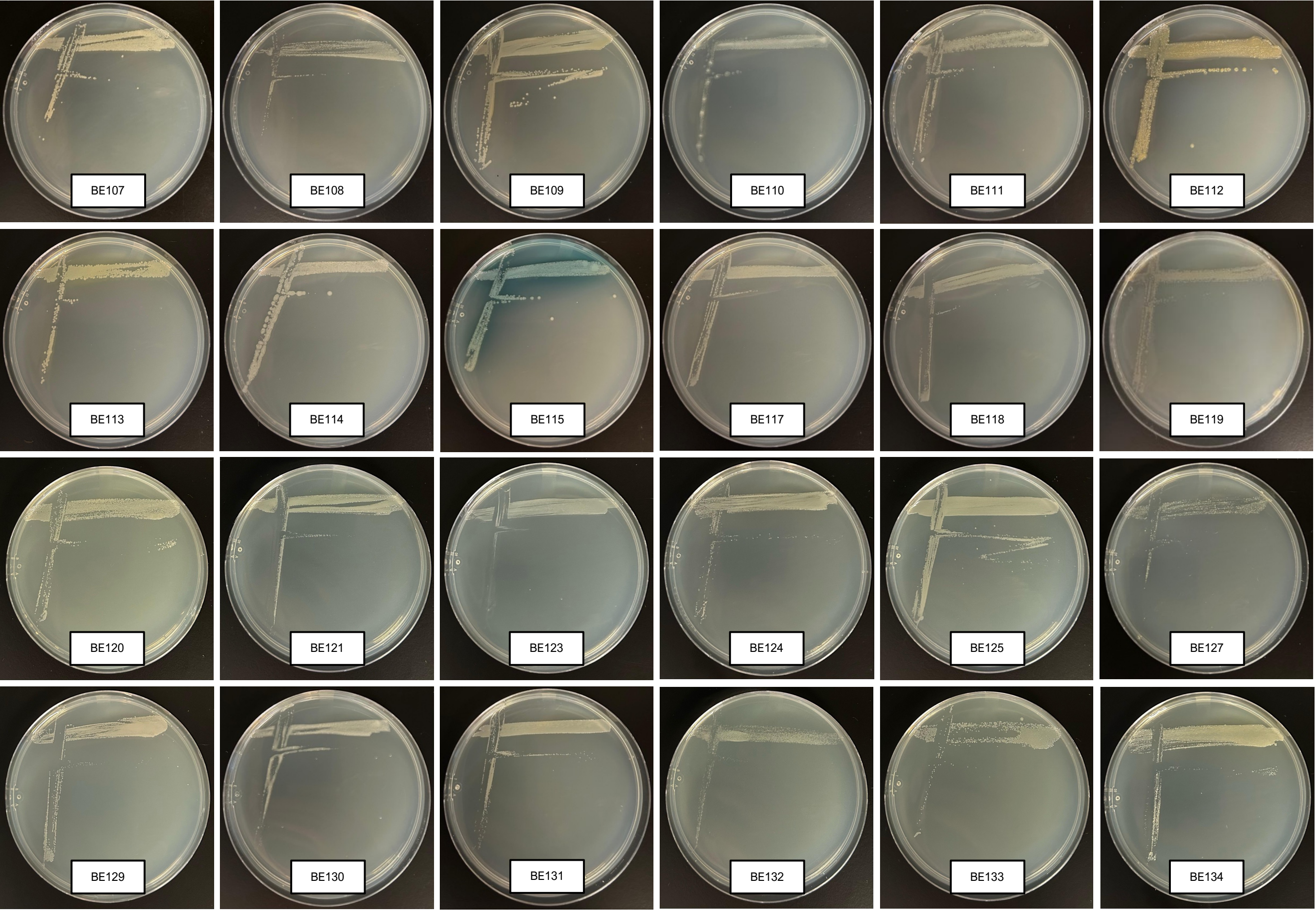

Figure S1. Photographs of the DPB *P. aeruginosa* strains utilized in this study. Varying phenotypes regarding color, transparency, colony size, colony morphology, and mucoidy are observed in the sample set.
