# Supplementary material for: Characterization of Pseudomonas aeruginosa from subjects with diffuse panbronchiolitis
Source: Microbiol Spectr. 2024 Oct 8;12(11):e00530-24. doi: 10.1128/spectrum.00530-24 (PMC11537112; doi:10.1128/spectrum.00530-24)
Supplement: Figure S3 — Lipid A mass spectra of each DPB P. aeruginosa strain in the sample set (BE107-BE134). [file spectrum.00530-24-s0003.docx]

**Supplemental Figures**

**Characterization of *Pseudomonas aeruginosa* from subjects with diffuse panbronchiolitis**

Charles M. Met^1^*, Casey E. Hofstaedter^1,2^*, Ian P. O’Keefe^1,3^, Hyojik Yang^1^, Dina A. Moustafa^4^, Matthew E. Sherman^1^, Yohei Doi^5^, David A. Rasko^1,6^, Charles R. Sweet^7^, Joanna B. Goldberg^4^, Robert K. Ernst^1^

1 - Department of Microbial Pathogenesis, University of Maryland – Baltimore, Baltimore MD, 21201, USA

2 - Medical Scientist Training Program, University of Maryland – Baltimore, Baltimore MD, 21201, USA

3 - Department of Biochemistry and Molecular Biology, University of Maryland – Baltimore, Baltimore MD, 21201, USA

4 - Department of Pediatrics, Division of Pulmonary, Asthma, Cystic Fibrosis, and Sleep, Emory University School of Medicine, Atlanta GA, 30322, USA

5 - Department of Medicine, University of Pittsburgh School of Medicine, Pittsburgh, PA 15213, USA.

6 – Institute for Genome Sciences, Department of Microbiology and Immunology, University of Maryland - Baltimore, MD, 21201, USA

7 - Chemistry Department, United States Naval Academy, Annapolis, MD 21402, USA

*Charles M. Met and Casey E. Hofstaedter contributed equally to this work.


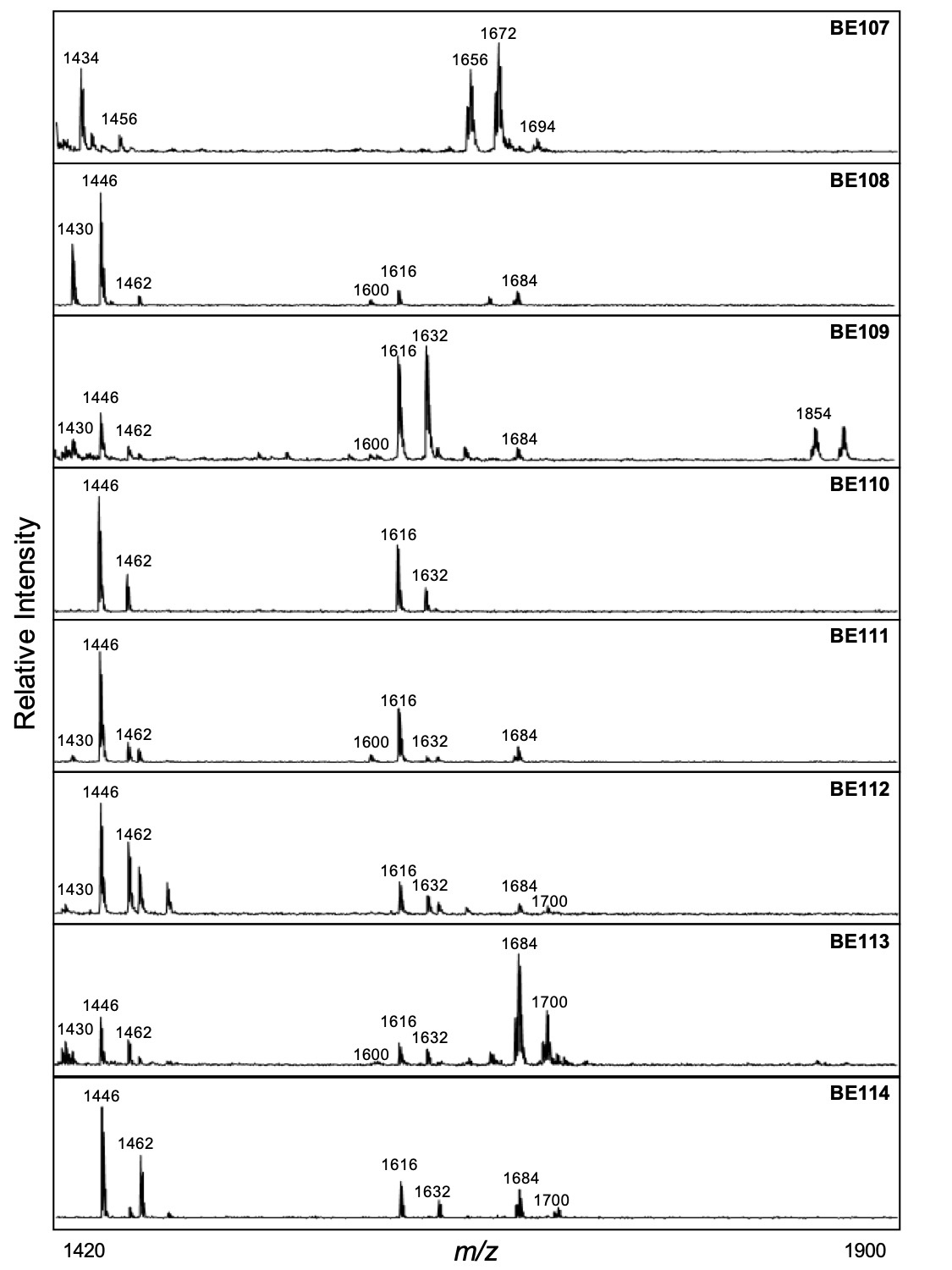

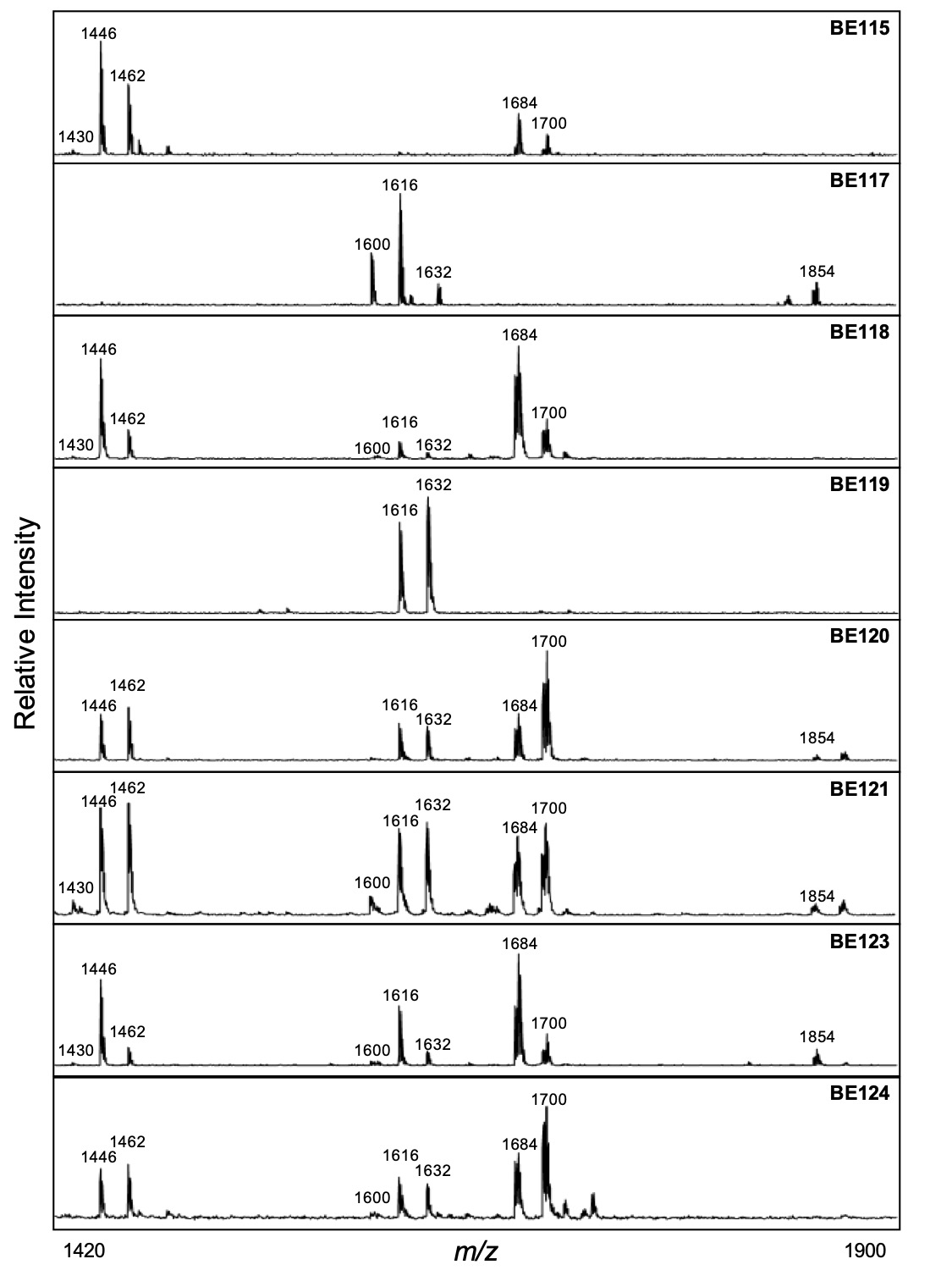

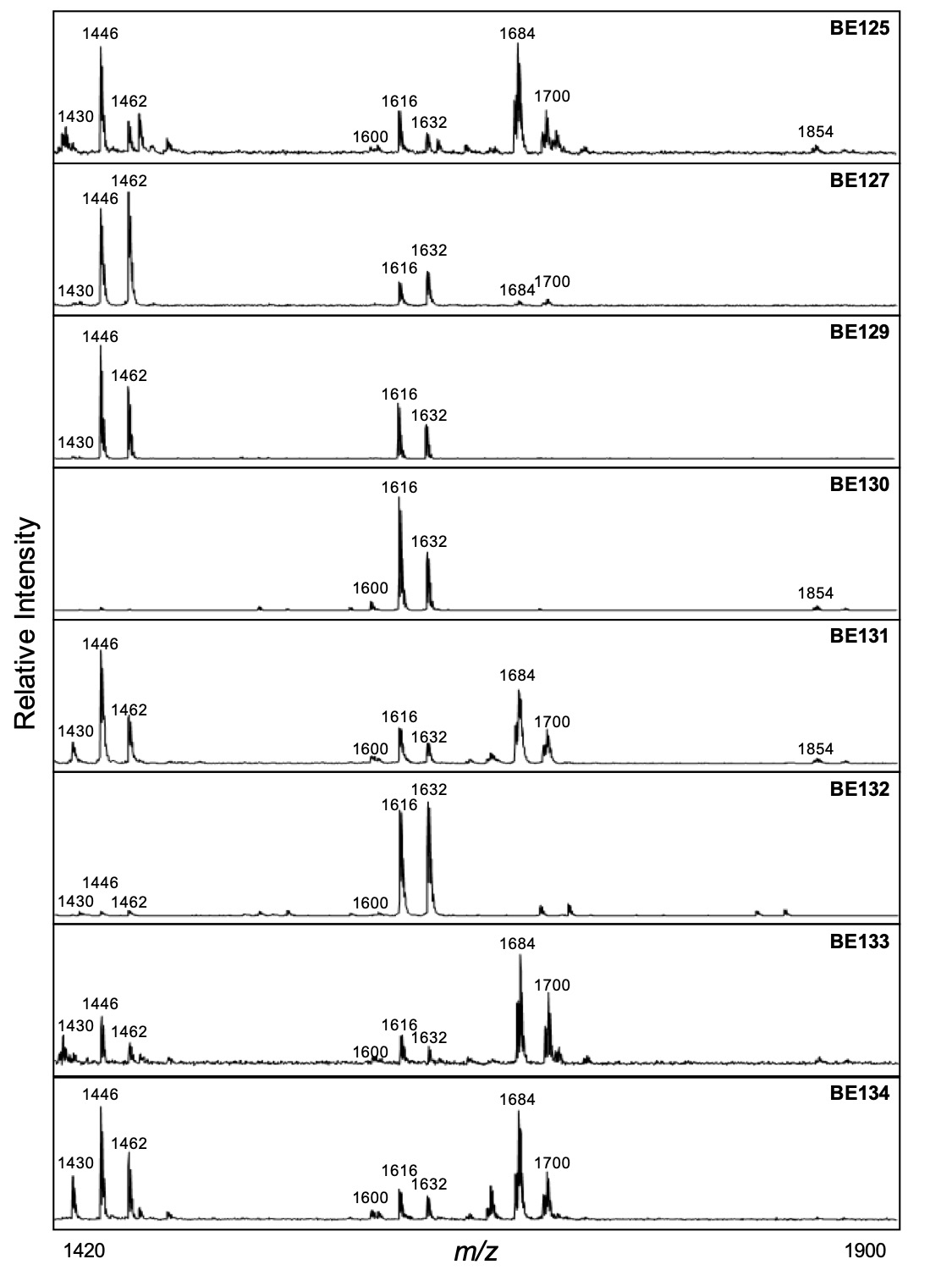


**A**

**B**

**Figure S3.** (**A**) Lipid A mass spectra of each DPB P. aeruginosa strain in the sample set (BE107-BE134). Lipid A peaks are labeled, located at m/z 1430, m/z 1446, m/z 1462, m/z 1600, m/z 1632, m/z 1684, m/z 1700, and m/z 1854. (**B**) Principal Component Analysis (PCA) of lipid A mass spectra based on presence or absence of lipid A peaks. PC1 and PC2 are shown, with 42.86% and 22.24% of the variance explained, respectively. Ellipses group strains based on observed lipid A structures, which are labeled in the graph. Strains that cluster together may overlap.
