# Supplementary material for: Characterization of Pseudomonas aeruginosa from subjects with diffuse panbronchiolitis
Source: Microbiol Spectr. 2024 Oct 8;12(11):e00530-24. doi: 10.1128/spectrum.00530-24 (PMC11537112; doi:10.1128/spectrum.00530-24)

**Supplemental Figures**

**Characterization of *Pseudomonas aeruginosa* from subjects with diffuse panbronchiolitis**

Charles M. Met^1^*, Casey E. Hofstaedter^1,2^*, Ian P. O’Keefe^1,3^, Hyojik Yang^1^, Dina A. Moustafa^4^, Matthew E. Sherman^1^, Yohei Doi^5^, David A. Rasko^1,6^, Charles R. Sweet^7^, Joanna B. Goldberg^4^, Robert K. Ernst^1^

1 - Department of Microbial Pathogenesis, University of Maryland – Baltimore, Baltimore MD, 21201, USA

2 - Medical Scientist Training Program, University of Maryland – Baltimore, Baltimore MD, 21201, USA

3 - Department of Biochemistry and Molecular Biology, University of Maryland – Baltimore, Baltimore MD, 21201, USA

4 - Department of Pediatrics, Division of Pulmonary, Asthma, Cystic Fibrosis, and Sleep, Emory University School of Medicine, Atlanta GA, 30322, USA

5 - Department of Medicine, University of Pittsburgh School of Medicine, Pittsburgh, PA 15213, USA.

6 – Institute for Genome Sciences, Department of Microbiology and Immunology, University of Maryland - Baltimore, MD, 21201, USA

7 - Chemistry Department, United States Naval Academy, Annapolis, MD 21402, USA

*Charles M. Met and Casey E. Hofstaedter contributed equally to this work.


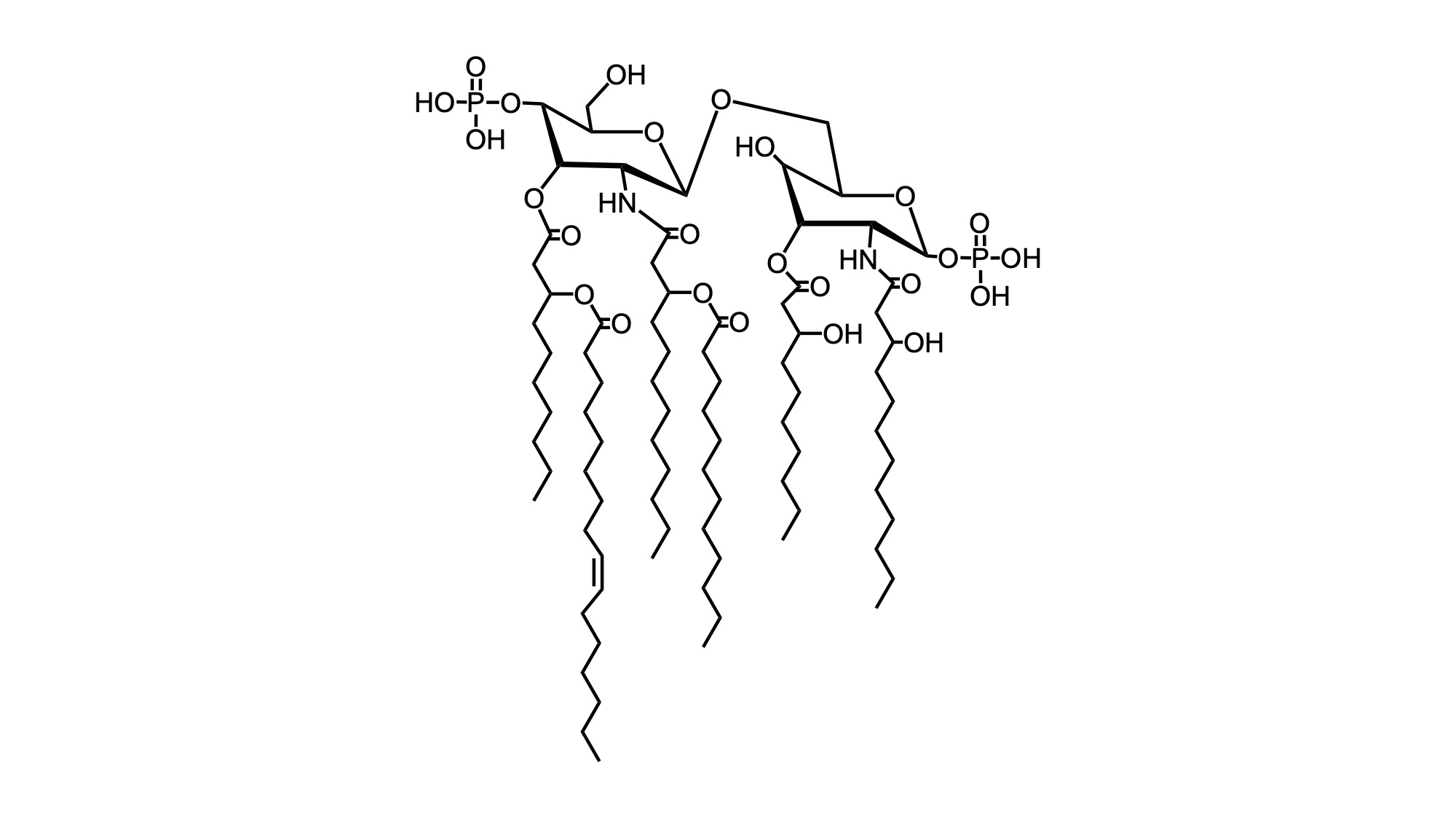

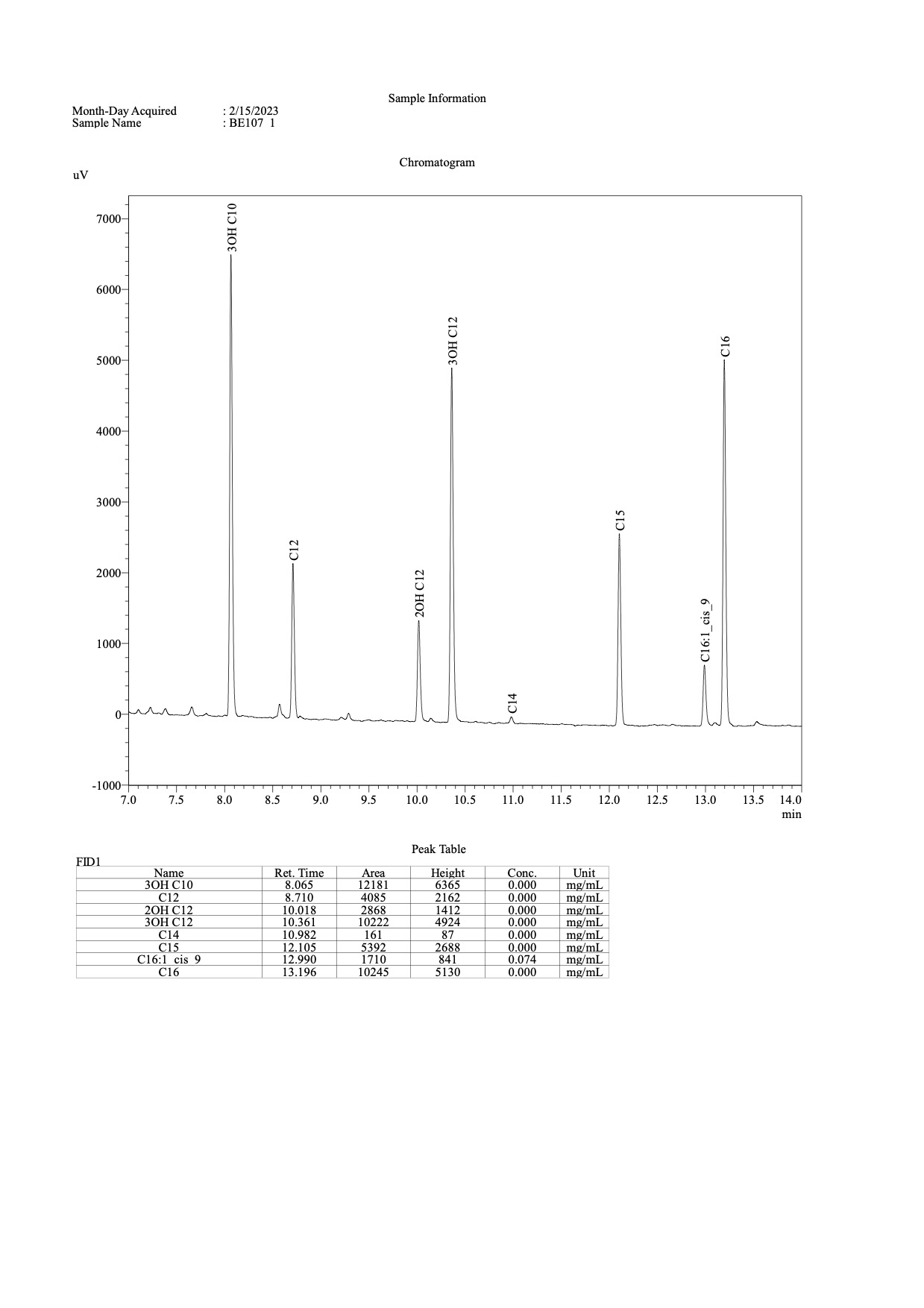


**C**

**B**

**A**


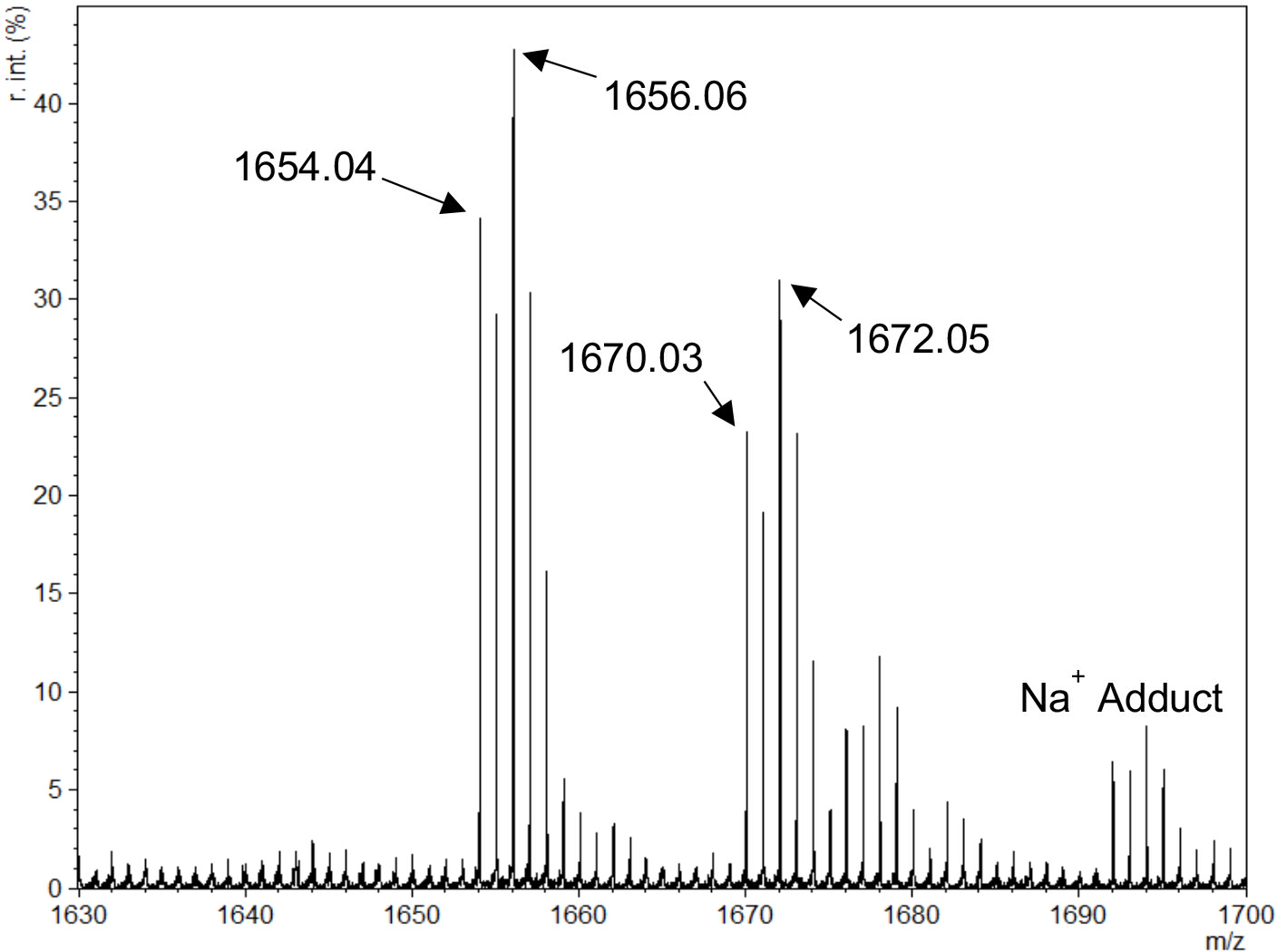
s

**Figure S4. Structural analysis of BE107. (A)** MS/MS data used to elucidate the novel BE107 lipid A structure. **(B)** GC-FID chromatogram of BE107 lipid A used to support the MS/MS data. **(C)** Proposed lipid A structure for BE107.

**B**


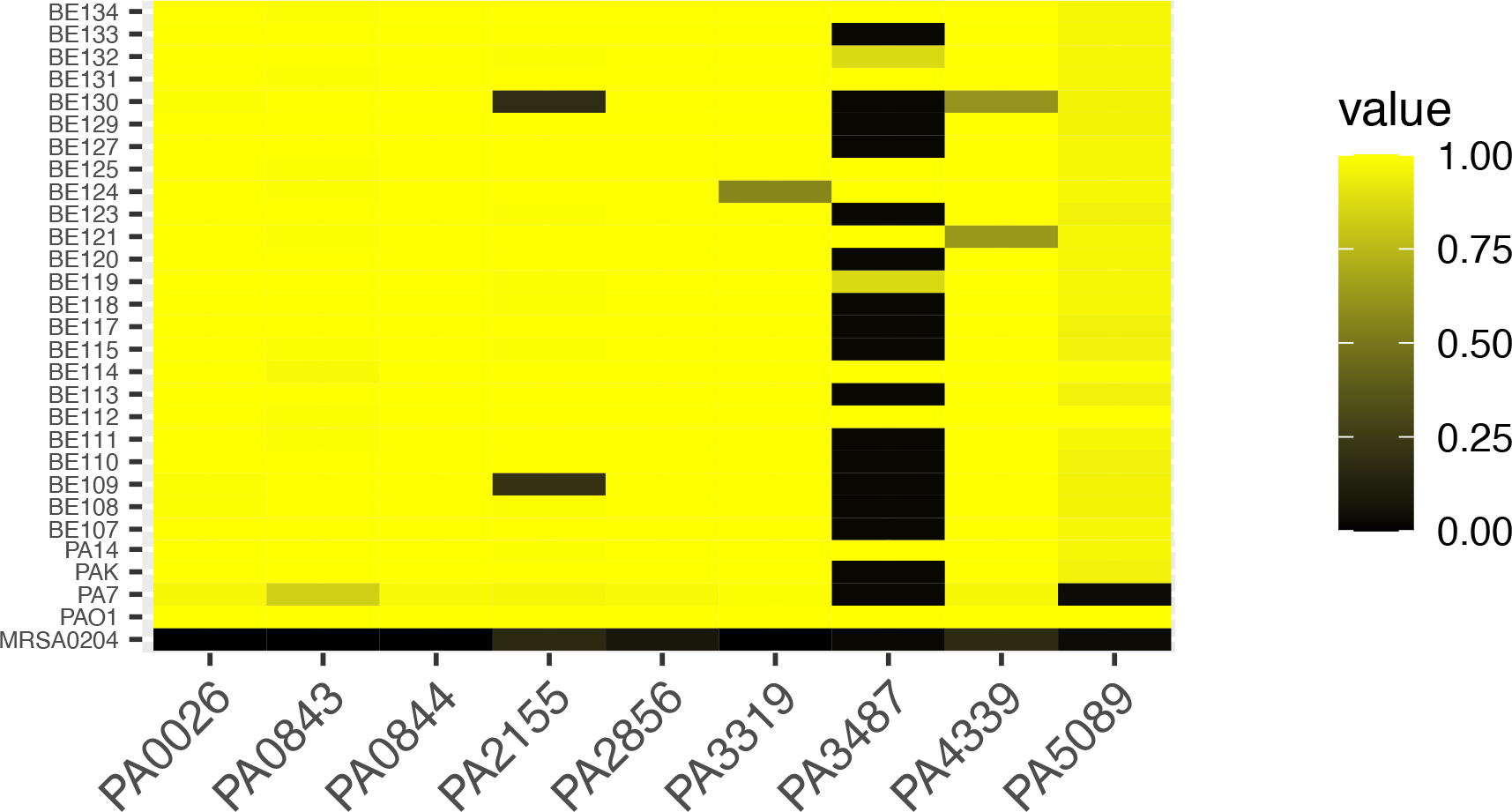

Supplement: Figure S4 — Structural analysis of BE107. [file spectrum.00530-24-s0004.docx]
