# Supplementary material for: Characterization of Pseudomonas aeruginosa from subjects with diffuse panbronchiolitis
Source: Microbiol Spectr. 2024 Oct 8;12(11):e00530-24. doi: 10.1128/spectrum.00530-24 (PMC11537112; doi:10.1128/spectrum.00530-24)
Supplement: Supplemental tables — Tables S2 to S5. [file spectrum.00530-24-s0006.docx]

**Supplemental Tables**

**Characterization of *Pseudomonas aeruginosa* from subjects with diffuse panbronchiolitis**

Charles M. Met^1^*, Casey E. Hofstaedter^1,2^*, Ian P. O’Keefe^1,3^, Hyojik Yang^1^, Dina A. Moustafa^4^, Matthew E. Sherman^1^, Yohei Doi^5^, David A. Rasko^1,6^, Charles R. Sweet^7^, Joanna B. Goldberg^4^, Robert K. Ernst^1^

1 - Department of Microbial Pathogenesis, University of Maryland – Baltimore, Baltimore MD, 21201, USA

2 - Medical Scientist Training Program, University of Maryland – Baltimore, Baltimore MD, 21201, USA

3 - Department of Biochemistry and Molecular Biology, University of Maryland – Baltimore, Baltimore MD, 21201, USA

4 - Department of Pediatrics, Division of Pulmonary, Asthma, Cystic Fibrosis, and Sleep, Emory University School of Medicine, Atlanta GA, 30322, USA

5 - Department of Medicine, University of Pittsburgh School of Medicine, Pittsburgh, PA 15213, USA.

6 – Institute for Genome Sciences, Department of Microbiology and Immunology, University of Maryland - Baltimore, MD, 21201, USA

7 - Chemistry Department, United States Naval Academy, Annapolis, MD 21402, USA

*Charles M. Met and Casey E. Hofstaedter contributed equally to this work.


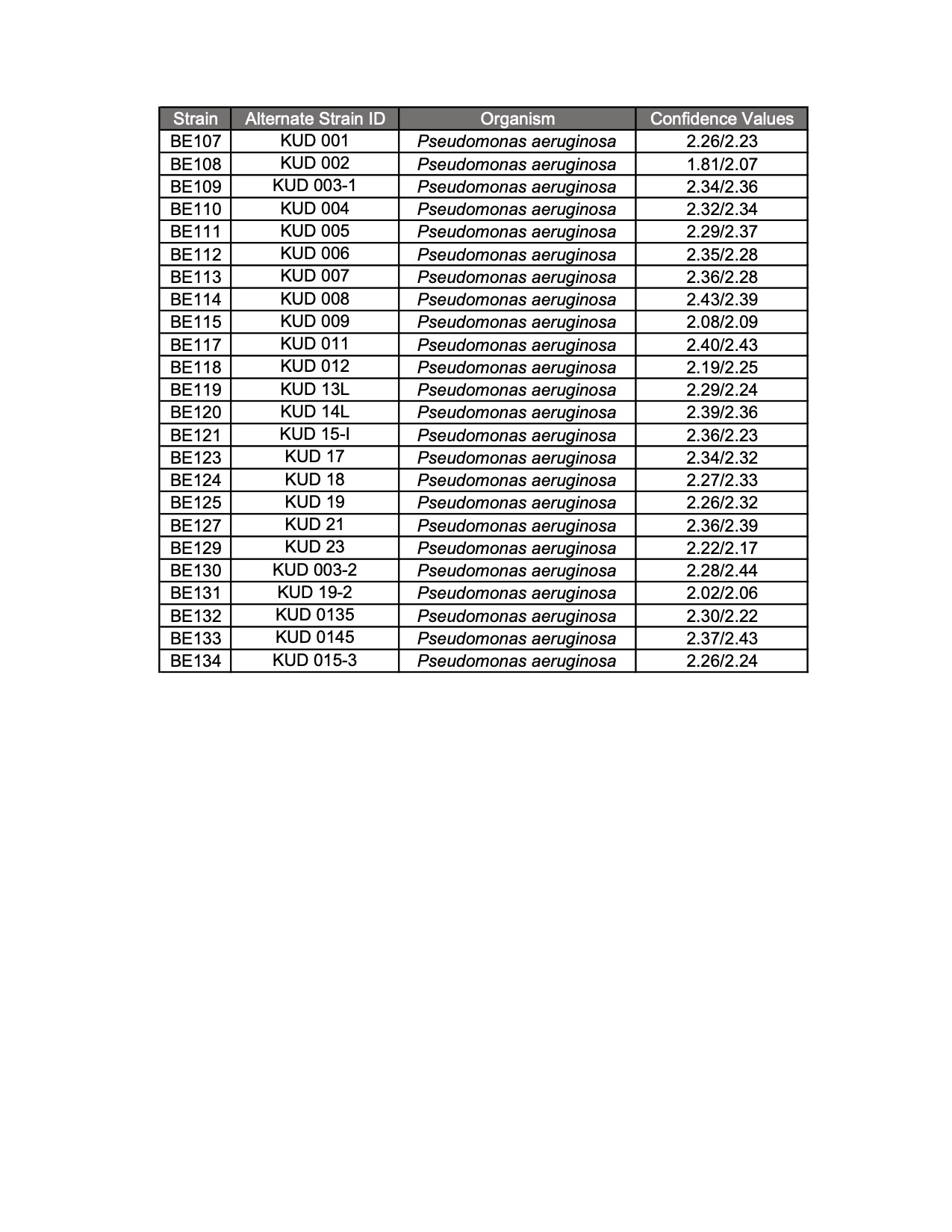


**Table S2. DPB P. aeruginosa microbial identification via MALDI Biotyper®.** Two bacterial spots, collected from two separate bacterial colonies, were tested per suspected DPB P. aeruginosa strain. Alternate strain identification labels are included for reference.

**Table S3. Genes more prevalent in the DPB genomes.** Using comparative genomics, we identified a suite of genes that are more prevalent among the DPB genomes when compared to the other isolates included (**Table S1**). This comparison was completed with Roary/Scoary and only those genes that had a Bonferroni corrected p-value < 0.0001 and were present in more than half of the DPB strains were included.

**Table S4. Tabulated summary of the DPB P. aeruginosa swim and swarm diameters.** Numerical values for swim motility represent haze diameters in millimeters (mm). Motility values are color coded based on the following criteria: orange <5 millimeters, blue 5-20 millimeters, and green >20 millimeters.

**Table S5. Summary table of P. aeruginosa O-specific antigen reactivity.** Western blot images are present in Appendix Figures 6, 7, and 8. The majority of DPB P. aeruginosa strains (19/24) did not have detectible O-antigen reactivity, suggesting these strains do not express O-antigen and have rough LPS.
