# Supplementary material for: Characterization of Pseudomonas aeruginosa from subjects with diffuse panbronchiolitis
Source: Microbiol Spectr. 2024 Oct 8;12(11):e00530-24. doi: 10.1128/spectrum.00530-24 (PMC11537112; doi:10.1128/spectrum.00530-24)
Supplement: Supplemental materials — Supplemental methods. [file spectrum.00530-24-s0007.docx]

**Supplemental Methods**

**Bacterial species identification**. Bruker MALDI Biotyper® was used according to manufacturer’s instructions. Bacteria were grown on LB agar plates for 18 hours at 37°C. Bacteria were then scraped onto a steel target plate in duplicate with a sterile toothpick. 1 µL of 70% formic acid was pipetted over bacterial spots. After drying, 1 µL of HCCA matrix was pipetted over bacterial spots. Bacterial identifications were made via a MALDI Biotyper® Sirius system (US-IVD). The MALDI Biotyper® Sirius system (US-IVD) produces a confidence score for each identification, ranging from no organism, low confidence, and high confidence identification. Scores below 1.7 indicate no organism identification, scores between 1.7 and 1.99 indicate low confidence identification, and scores of 2.0 and higher indicate high confidence identification.

**Preparation of genomic DNA.** Genomic DNA was isolated from aerobic culture grown at 37°C in LB using a GenElute bacterial genomic DNA kit (Sigma-Aldrich, St. Louis, MO). DNA concentrations were determined using a NanoDrop 1000 spectrophotometer (ThermoFischer Scientific, Waltham, MA).

**Swim motility.** Swim motility was assessed as previously described.^1^ The following media recipe was used: proteose-peptone (0.5%), yeast extract (0.3%), and agar (0.3%). Media was then added to Petri dishes. Over the center of each petri dish, 2 µL of liquid bacterial culture (grown in LB +1 mM MgCl_2_) were spotted. Plates were incubated without inversion and haze diameters were measured in centimeters. Assays were performed in biological triplicate.

**Swarm motility.** The following media recipe was used: proteose-peptone (0.5%), yeast extract (0.3%), and agar (0.5%). Media was then added to Petri dishes. Over the center of each petri dish, 2 µL of liquid bacterial culture (grown in LB +1mM MgCl_2_) were spotted. Plates were incubated without inversion and swarm diameters were measured in centimeters. Assays were performed in biological triplicate.

**Bacterial growth curves.** Liquid bacterial cultures grown for 18 hours in LB were standardized to 0.5 McFarland standard (1x10^8^ CFU/mL) with LB using a DensiCHEK Plus (BioMerieux, Salt Lake City, UT). Cultures were then diluted 1:20 in LB. In a 96-well plate, 10 µL of culture was added to 90 µL of fresh media prior to data collection. The 96-well plate was incubated in a Stratus plate reader (Cerillo, Charlottesville, VA). OD600 measurements were recorded every 15 minutes for 24 hours. Assays were performed in biological triplicate.

**Genome-based comparisons.**

Roary/Scoary: All genomes used in subsequent analyses (Tables S1a/S1b) were annotated via Prodigal^2^ to generate *de novo* CDS then PROKKA v1.14.6.^3^ The resulting General Feature Formats (GFF) were analyzed to identify core and accessory genes using Roary v3.13.0^4^ and Scoary version 1.6.16.^5^

**Tandem mass spectrometry (MS/MS).** Single colonies were smeared onto ITO-glass slides. FLAT extraction buffer (1 µL; 0.2 M anhydrous citric acid, 0.1 M trisodium citrate dihydrate) was pipetted over bacterial spots. The ITO-glass slides were incubated in a “Panini-Press” heat block at 100°C for 30 minutes. Bacterial spots were washed several times with ddH_2_O and air-dried, over which 1 µL of 10 mg/mL Norharmane matrix was pipetted. Samples were processed with a Bruker MALDI-TOF MS and a Thermo MALDI Linear Ion Trap (LTQ)-XL-Orbitrap (San Jose, CA). The Bruker MALDI-TOF MS was equipped with a dual ESI/MALDI source with a Smart Beam 3D 10 KHz frequency tripled Nd:YAG laser (355 nm). The system was operated in qTOF mode (TIMS deactivated). Ion transfer tuning was used with the following parameters: Funnel 1 RF 440.0 Vpp, Funnel 2 RF 490.0 Vpp, Multipole RF 490.0 Vpp, CID energy 0.0 eV, and Deflection Delta 60.0 V. Quadrupole was used with Ion Energy 4.0 eV and Low Mass 700.00 *m/z*. Collision cell activation of ions used Collision Energy 9.0 eV and Collision RF 3900.0 Vpp. The MS/MS precursor ion was chosen by targeting *m/z* values two digits to the right of the decimal point. Isolation width was set to 4-6 *m/z* and collision energy was set to 100-110 eV. Focus Pre TOF used a Transfer Time of 110.0 µs and Pre-Pulse Storage of 9.0 µs. Agilent ESI Tune Mix was used to calibrate the machinery. Mass spectra were collected in negative-ion mode with a 104 µm laser diameter using 800 laser shots at 70-80% laser power. Mass spectra were analyzed with mMass (Version 5.5.0) and ChemDraw Ultra (Version 10.0) software.

**Gas chromatography with flame-ionization detection (GC-FID).** Bacterial cultures (50 mL) were centrifuged at 6000 rpm for 10 minutes and the supernatant was discarded. Bacterial pellets were resuspended in 500 µL ddH_2_O and 500 µL 90% phenol. Samples were incubated at 70°C for 1 hour with agitation. Samples were cooled over ice for 5 minutes and centrifuged at 10,000 rpm for 10 minutes. The aqueous phases were transferred to screw cap microcentrifuge tubes. 500 µL of water was added to each sample and the 70°C incubation through the aqueous phase collection steps were repeated two additional times. Samples were washed with 2 mL of diethyl ether and centrifuged at 3000 rpm for 5 minutes. Aqueous phases were collected and transferred to gas chromatography vials. Samples were exposed to dry ice for 20 minutes and lyophilized overnight. Then, 50µL of 2 mg/mL C15 internal standard and 200 µL 2 M methanolic HCl were added to the samples. Each sample was incubated in a 90°C heat block overnight, after which 200 µL of NaCl saturated water and 400µL of hexane was added to the samples. The Hexane layers were transferred to gas chromatography vials and the addition of 400 µL hexane through the hexane layer collection steps were repeated twice. Samples were processed using a GC-2010 Plus GC for Mass Spectrometer (Shimadzu, Torrance, CA). Spectra were analyzed on GC Postrun Analysis (Shimadzu, Torrance, CA) and ChemDraw Ultra (Version 10.0) software. Assays were performed in biological triplicate.

**Antibiotic Susceptibility Testing.** Antibiotic susceptibility testing (AST) was performing using a Kirby-Bauer disk diffusion assay. Bacteria grown in LB for 18hrs were standardized to 0.5 McFarland standard (1x10^8^ CFU/mL) using a MicroScan Turbidity Meter (Beckman Coulter, Brea, CA). Cotton swabs soaked in culture were used to plate a bacterial lawn on Müller Hinton agar. Antibiotic disks were placed flush to the agar surface prior to incubation. Plates were incubated at 37°C for 24 hours, and zone of inhibition diameters were measured in millimeters.

***Pseudomonas aeruginosa* serotyping.** LPS was purified from *P. aeruginosa* overnight cultures grown on LB agar and were resuspended in PBS and normalized to OD_600_ = 0.5 (~1x10^9^ CFU / mL). Washed bacteria was then pelleted by centrifugation and resuspended in 200µl SDS sample buffer (BioRad) and briefly boiled. Samples were incubated with DNaseI (240 µg/mL), RNase (240 µg/mL) for 30 minutes at 37°C, followed by 3 hours incubation with Proteinase K (465 µg/mL) at 59°C. Ten µl purified LPS was loaded and separated on a 12% Tris-glycine gel (BioRad) using SDS-PAGE and visualized by Western blotting. *P. aeruginosa* serogroup rabbit polyvalent antibodies (Denka Seiken, Tokyo Japan) were used for Western blotting. Anti-rabbit IgG-HRP was used as a secondary antibody, respectively.

References

1. Ha DG, Kuchma SL, O’Toole GA. Plate-Based Assay for Swimming Motility in Pseudomonas aeruginosa. In: ; 2014:59-65. doi:10.1007/978-1-4939-0473-0_7

2. Hyatt D, Chen GL, Locascio PF, Land ML, Larimer FW, Hauser LJ. *Prodigal: Prokaryotic Gene Recognition and Translation Initiation Site Identification*.; 2010. http://www.biomedcentral.com/1471-2105/11/119

3. Seemann T. Prokka: Rapid prokaryotic genome annotation. *Bioinformatics*. 2014;30(14):2068-2069. doi:10.1093/bioinformatics/btu153

4. Page AJ, Cummins CA, Hunt M, et al. Roary: Rapid large-scale prokaryote pan genome analysis. *Bioinformatics*. 2015;31(22):3691-3693. doi:10.1093/bioinformatics/btv421

5. Brynildsrud O, Bohlin J, Scheffer L, Eldholm V. Rapid scoring of genes in microbial pan-genome-wide association studies with Scoary. *Genome Biol*. 2016;17(1). doi:10.1186/s13059-016-1108-8
